# Highly efficient production of rebaudioside D enabled by structure-guided engineering of bacterial glycosyltransferase YojK

**DOI:** 10.3389/fbioe.2022.985826

**Published:** 2022-08-25

**Authors:** Baodang Guo, Xiaodong Hou, Yan Zhang, Zhiwei Deng, Qian Ping, Kai Fu, Zhenbo Yuan, Yijian Rao

**Affiliations:** ^1^ Key Laboratory of Carbohydrate Chemistry and Biotechnology, Ministry of Education, School of Biotechnology, Jiangnan University, Wuxi, China; ^2^ School of Life Sciences and Health Engineering, Jiangnan University, Wuxi, China

**Keywords:** rebaudioside D, rebaudioside A, YojK, glycosyltransferase, structure-guide engineering, cascade reaction

## Abstract

Owing to zero-calorie, high-intensity sweetness and good taste profile, the plant-derived sweetener rebaudioside D (Reb D) has attracted great interest to replace sugars. However, low content of Reb D in *stevia rebaudiana* Bertoni as well as low soluble expression and enzymatic activity of plant-derived glycosyltransferase in Reb D preparation restrict its commercial usage. To address these problems, a novel glycosyltransferase YojK from *Bacillus subtilis* 168 with the ability to glycosylate Reb A to produce Reb D was identified. Then, structure-guided engineering was performed after solving its crystal structure. A variant YojK-I241T/G327N with 7.35-fold increase of the catalytic activity was obtained, which allowed to produce Reb D on a scale preparation with a great yield of 91.29%. Moreover, based on the results from molecular docking and molecular dynamics simulations, the improvement of enzymatic activity of YojK-I241T/G327N was ascribed to the formation of new hydrogen bonds between the enzyme and substrate or uridine diphosphate glucose. Therefore, this study provides an engineered bacterial glycosyltransferase YojK-I241T/G327N with high solubility and catalytic efficiency for potential industrial scale-production of Reb D.

## Introduction

Due to the consumption of high-sugar diets, more and more people suffer from different chronic diseases, such as obesity, diabetes, cardiovascular diseases, and hypertension, which have become a serious global public health problem (Xu et al., 2013; Wu et al., 2018; Plaza-Diaz et al., 2020). Therefore, it is of great interest to exploit the new generation of sweeteners with low- or zero-calorie. Plant-derived steviol glycosides with high-intensity sweetness have been recognized as low- or zero-calorie sweeteners to replace high-calorie sugars because they are basically not absorbed and metabolized after intake by human body (Gardana et al., 2003; Kurek et al., 2020). Over 60 types of steviol glycosides, including stevioside, rebaudioside A (Reb A), rebaudioside D (Reb D) and rebaudioside M (Reb M), have been isolated and identified from leaves of *Stevia rebaudiana* Bertoni (Espinoza et al., 2014; Guo et al., 2019). The major steviol glycosides in *S. rebaudiana* Bertoni are stevioside (5%–10% of leaf dry weight) and Reb A (2%–4% of leaf dry weight) (Goyal et al., 2010; Wang et al., 2021). They exhibit 250–300 times higher sweetness than sucrose but with an annoying bitter aftertaste (Hellfritsch et al., 2012). Compared with stevioside and Reb A, Reb D and Red M have a much less lingering bitter aftertaste (Hellfritsch et al., 2012). Similarly, their sweetness are also much higher than sucrose, up to 350 times. These properties make them as the new-generation of natural sweeteners (Prakash et al., 2014c). However, they are less abundant, approximately 0.4%–0.5% of leaf dry weight (Ceunen and Geuns, 2013). Thus, traditional extraction for *stevia* plants for commercial usage of them is impractical. As Reb D can be biologically converted from Reb A by glycosyltransferase UGT91D2 (Zhang et al., 2021), which can be further used as the substrate for biosynthesis of Reb M by another glycosyltransferase UGT76G1 in plant *S. rebaudiana* Bertoni (Liu et al., 2020), the enzymatic preparation of Reb D from Reb A on a commercial scale has been considered as an economically effective method and attracted widespread attention.

To date, substantial efforts focused on the identification of new glycosyltransferases with the catalytic ability to glycosylate Reb A to synthesize Reb D. Besides UGT91D2 from the original biosynthesis pathway of Reb D with low substrate specificity (Zhang et al., 2021), glycosyltransferases UGTSL2 from *Solanum lycopersicum* and EUGT11 from *Oryza sativa* show the biocatalytic ability of the conversion of Reb A to produce Reb D (Prakash et al., 2014a; Prakash et al., 2014b; Chen et al., 2018; Wang et al., 2020; Wang et al., 2021). With the recycle of uridine diphosphate glucose (UDPG) catalyzed by sucrose synthase StSUS1, Reb D can be efficiently biosynthesized by UGTSL2, but with side products of Reb D2 and Reb M2 (Prakash et al., 2014a; Prakash et al., 2014b; Chen et al., 2018). The Asn358Phe mutant of UGTSL2 can increase its catalytic activity with less side products (Chen et al., 2020). EUGT11 also shows its catalytic ability to biosynthesize Reb D from Reb A at the low concentration of Reb A (Wang et al., 2020). However, the heterologous expression of these plant-derived glycosyltransferases has been widely recognized as a problem (Cai et al., 2017; Chen et al., 2018; Lemmerer et al., 2019; Shu et al., 2020). They are mainly expressed as inclusion bodies in *Escherichia coli* (*E. coli*) with little soluble and active protein (Cai et al., 2017; Lemmerer et al., 2019; Shu et al., 2020), which greatly restricts their industrial applications for scale production of Reb D. Therefore, to identify and characterize a novel glycosyltransferase, which could be functionally expressed in *E. coli* with high solubility and possesses an excellent ability to catalyze the synthesis of Reb D from Reb A with great regioselectivity, is highly desired.

Here we report a novel glycosyltransferase YojK from *Bacillus subtilis* 168 (*B. subtilis* 168) with the catalytic ability to glycosylate Reb A to produce Reb D. This glycosyltransferase YojK could be functionally expressed in *E. coli* BL21 (DE3) with excellent solubility, which allowed us to solve its crystal structure. Based on its crystal structure, we then analyzed its catalytic mechanism and performed structure-guided engineering. Then, a double mutant YojK-I241T/G327N, which greatly improved the catalytic activity with 7.35 folds increase compared with wild-type YojK, was identified. After optimization of reaction conditions, 20.59 g/L Reb D with an excellent yield of 91.29% was produced on a scale preparation from 19.32 g/L (20 mM) Reb A after 15 h in cascade reactions containing YojK-I241T/G327N and sucrose synthase *At*SuSy used for the recycle of UDPG. Thus, these advantages of YojK-I241T/G327N variant provide the potential possibility for industrial scale production of Reb D.

## Materials and methods

### Strains, plasmids and chemicals

The strains and plasmids used in this study are listed in Supplementary Table S1. YojK from *B. subtilis* 168 (accession numbers: CP053102) and *AtSuSy* from *Arabidopsis thaliana* (accession numbers: NM_001036838.2) were codon-optimized and synthesized by Exsyn-bio (Wuxi, China). *E. coli* (*E. coli*) Top10 was used for constructing plasmids. pET-21b (+) and *E. coli* BL21 (DE3) were used for the protein expression of YojK or its variants, while pACYCDuet-1 was used for the co-expression of YojK and *At*SuSy for the cascade reaction. The gene of YojK was inserted into the pET-21b (+) under the T7 promoter using a seamless cloning and assembly kits from Exsyn-bio (Wuxi, China). Mutations of YojK were generated by polymerase chain reaction (PCR) with the corresponding primers as shown in Supplementary Table S2. All insertions and mutations of target genes were sequenced by Genewiz (Suzhou, China). Authentic samples of Reb A and Reb D were purchased from Macklin Biotechnology (China). All other reagents were purchased from Sangon Biotech (Shanghai, China).

### Protein expression and purification

All plasmids used for protein expression were transformed into *E. coli* BL21 (DE3) supplemented with appropriate antibiotics. Then, positive clones were inoculated into 5 ml LB medium with appropriate antibiotics to prepare overnight culture, which were further inoculated into 1 L TB medium supplemented with appropriate antibiotics in a baffled flask at ratio of 1:100. 0.1 mM isopropyl-β-D-thiogalactopyranoside (IPTG) was added to induce protein expression at 18°C for 8 h when OD_600_ reached 0.6–0.8.

The cells were harvested by centrifugation at 7,000 × *g* for 7 min and then resuspended in lysis buffer (50 mM Tris-HCl pH 8.0, 300 mM NaCl, 10 mM imidazole and 10% glycerol) at the final concentration of 0.1 g/ml. The cells were disrupted using a high-pressure homogenizer (Union-Biotech Co., Ltd., Shanghai, China). Cell debris was immediately removed by centrifugation at 40,000 × *g* for 30 min. Target proteins were purified by a Ni-NTA column from supernatants. The eluted protein samples were loaded onto a Superdex 200 column (GE Healthcare, Pittsburgh, United States) for further purification with 25 mM Tris-HCl pH 8.0 and 150 mM NaCl as running buffer. The purified proteins were analyzed by sodium dodecyl sulfate polyacrylamide gel electrophoresis (SDS-PAGE) and stored at −80°C or immediately used for enzymatic assays or crystallization.

### *In vitro* enzymatic assays

Typically, a 200 μl reaction system contained 4 mM Reb A, 10 μM YojK or its variants, 10 mM UDPG, 10 mM MnCl_2_, 50 mM Tris-HCl pH 8.0 was used to analyze the catalytic activity of YojK and its variants. The mixtures were then incubated at 35°C for 20 min and quenched with 5 folds volume of methanol. The resultant samples were centrifuged at 20,000 × *g* for 5 min to remove the protein precipitant. Then, the supernatants were filtered by 0.22 μm filters and loaded onto Waters Acquity UPLC system equipped with BEH C18 1.7 μM analytical column (2.1 mm × 50 mm) and detected at 210 nm for analysis. The mobile phase was acetonitrile with 1.38 g/L NaH_2_PO_4_ buffer (pH 2.6). The solvent flow rate was 0.3 ml/min and the column temperature was 40°C. The elution program was as follows: 0–1min: 15% acetonitrile isocratic elution, 1–2 min: 15%–27% acetonitrile linear gradient elution, 2–6 min 27%–33% acetonitrile linear gradient elution. All reactions were performed in triplicate. The standard curve of Reb D was employed to calculate the content of Reb D in the reaction (Supplementary Figure S1).

LC-MS analysis was performed in electrospray ionization negative mode with BEH C18 1.7 μM analytical column (2.1 mm × 50 mm) on a MALDI SYNAPT Q-TOF MS system (Waters, Massachusetts, United States). The mobile phase was acetonitrile with aqueous (0.1% formic acid), and the elution program was same as described above.

### Kinetic analysis

To detect the catalytic activity of YojK and its variants, the enzymatic activity assay was performed in 200 μl reaction buffer containing 5 mM UDPG, 10 mM MnCl_2_, 50 mM Tris-HCl pH 8.0, and 2.5–20 μg of purified protein samples (depended on the catalytic activity of YojK variants) with the concentration of Reb A ranging from 0.05 to 0.7 mM. The reaction was carried out at 35°C for 20 min and quenched with equal volume of methanol. Then the mixtures were prepared and analyzed as described in “*In vitro* enzymatic assays” section.

### Preparation and characterization of Reb D

Reb D was characterized using an Agilent 1200 preparative HPLC system coupled with a ZORBAX Eclipse XDB C18 column (9.4 mm × 250 mm, 5 μm, Angilent, United States) with UV detection at 210 nm. The mobile phase was the mixture of acetonitrile and H_2_O. The solvent flow rate was 3 ml/min and the column temperature was 40°C. The elution program was as follows: 0–30 min: 27% acetonitrile isocratic elution. 20 mg purified sample was dissolved with pyridine-*d*_*5*_ and then analyzed by a Bruker Avance III 600 MHz nuclear magnetic resonance (^1^H NMR, ^13^C NMR, COSY, TOCSY, HSQC, HMBC, ROESY) spectrometer (Bruker BioSpin, Karlsruhe, Germany).

### Crystallization, data collection and structure determination

Purified YojK with 5 mM uridine diphosphate (UDP) was concentrated to 10 mg/ml and used for crystal screening by sitting-drop vapor-diffusion method with different commercial crystallization screening kits from Hampton Research (State of California, United States). All experiments were performed at 18°C. YojK crystals were obtained by incubating 0.7 μl YojK-UDP complex (3 mg/ml) with 0.7 μl reservoir solution (0.09 M magnesium chloride, 0.09 M Tris-HCl, pH 8.0, 25.2% (w/v) PEG3350 and 0.05 M sodium fluoride). For data collection, the crystals were flash-frozen in liquid nitrogen with the reservoir solution containing 20% ethylene glycol as the cryoprotectant. The dataset were collected with CCD camera on BL-18U1 stations of the Shanghai Synchrotron Radiation Facility (SSRF).

Diffraction data were indexed, integrated and scaled by HKL2000 software package. The structure of YojK-UDP complex were solved by molecular replacement with crystal structure of YjiC (PDB ID: 6kqx) as a model. Later, manual model was built by COOT and refined with REFMAC to obtain the crystal structure of YojK. Data collection and refinement statistics were summarized in Supplementary Table S3. Atomic coordinates and structure factors have been deposited to Protein Data Bank (PDB) under accession number 7VM0.

### Molecular docking and molecular dynamics simulations

Based on crystal structure of YojK, the missing loops of original crystal structure were rebuilt using Modeller software (Webb and Sali, 2016). The UDP in crystal structure of YojK-UDP complex was replaced by UDPG to generate YojK-UDPG complex. Water molecules were removed from YojK-UDPG complex. Substrate Reb A was docked into YojK-UDPG complex using Discovery Studio 2016. The appropriate YojK-UDPG-Reb A conformations with lowest energy were used for molecular dynamic simulations.

All molecular dynamic simulations were performed using Amber18 with its GPU-accelerated pmemd module. The YojK-I241T/G327N was manually built using pyMOL, followed with a minimization, and assigned with ff14SB parameter field (Maier et al., 2015). Parameters for UDPG and Reb A were built using general Amber force field (GAFF) (Wang et al., 2004). The complex was solvated with a cubic TIP3P water molecule box with a least distance of 12 Å from protein to water box boundary. In order to neutralize the system, sodium ions were added. For the molecular dynamics (MD) simulations, a minimization using steepest descent algorithm (2,500 steps) and conjugate gradient algorithm (2,500 steps) was implemented with a restraint constant of 500 kcal mol^−1^ Å^2^ for YojK-UDPG-Reb A complex or YojK-I241T/G327N-UDPG-Reb A, and then followed by a same minimization without any restraint. The system was submitted to a heating procedure from 0 to 300 K in 200 ps. After that, an unrestrained 200 ps equilibration was implemented. Finally, a 200 ns MD production was conducted. During all simulations, the classical nonbonded cut-off was set to 10 Å, the integration step was set to 2 fs. For calculating long-range electrostatic interactions, the Particle Mesh Ewald (PME) method was adopted (Essmann et al., 1995). SHAKE algorithm was employed to constrain the bond length involving hydrogen atoms (Miyamoto and Kollman, 1992).

### Preparation of cell lysate for the cascade reaction

To prepare enough amount of cell lysates for the cascade reaction, protein co-expression was carried out with TB medium in a 7 L fermenter. Single clone was picked into 5 ml LB medium with 34 μg/ml chloramphenicol and incubated at 37°C for 12 h, which were then inoculated into a 7 L fermenter with TB medium containing 34 μg/ml chloramphenicol. When OD_600_ reached 0.6–0.8, 0.2 mM IPTG was added to induce protein expression at 18°C for 18 h. During the fermentation, ammonium hydroxide and phosphoric acid were used to retain pH at 6.86. Glycerol was added to keep the dissolved oxygen around 30%. The cells were harvested by centrifugation at 7,000 × *g* for 7 min and washed twice by 100 mM potassium phosphate (KPi) buffer pH 8.0. The pellet was resuspended with the buffer containing 100 mM KPi (pH 8.0) and 100 mM NaCl, and then disrupted using high-pressure homogenizer. Cell debris was immediately removed by centrifugation at 40,000 × *g* for 30 min. The protein concentration of supernatant was determined by Nano-Drop 2000 UV-Vis spectrophotometer with three repeats at 280 nm wavelength. Subsequently, the supernatant was used for the cascade reaction.

### Optimization of the YojK-I241T/G327N-*At*SuSy cascade reaction

Standard YojK-I241T/G327N-*At*SuSy cascade reaction system (1 ml) containing 100 mM KPi pH 8.0, 100 mM NaCl, 40 mg/ml crude enzyme, 20 mM Reb A, 200 mM sucrose, 10% DMSO (v/v), incubating at 35°C for 6 h, was used to optimize the reaction conditions. 100 mM Bis-Tris buffer pH 5.5-6.0 and 100 mM NaCl, 100 mM K_2_HPO_4_-KH_2_PO_4_ buffer pH 6.0-8.0 and 100 mM NaCl, 100 mM Tris-HCl buffer pH 8.0-9.0 and 100 mM NaCl were used to determine the optimal pH. Then, temperature (20°C–45°C), the ratio of DMSO (5%–25%) and the concentration of sucrose (0–800 mM) were analyzed with the optimal pH. Last, the optimization of the concentration of Reb A was investigated when the optimal temperature, the ratio of DMSO and concentration of sucrose were available. A scale reaction was carried out in 20 ml with additional 1 mM UDPG under the above optimized conditions. Samples were taken at different reaction time points for analysis. All reactions were performed in triplicate and corrected.

The yield of Reb D was determined as follows:

$$\text{The}\;\text{yield}\;\text{of}\;\text{Reb}\;\text{D}=C_{o}\left(\text{Reb}\;\text{D}\right)/C_{t}\left(\text{Reb}\;\text{D}\right)\times 100\%$$

*C*_*o*_ (Reb D) is the actual concentration of Reb D in the reaction system, and *C*_*t*_ (Reb D) is the theoretical concentration of Reb D in the reaction system. Reb D was quantified by using its standard curve.

## Results and discussion

### Glycosylation of Reb A to synthesize Reb D by glycosyltransferase YojK

**FIGURE 1:**
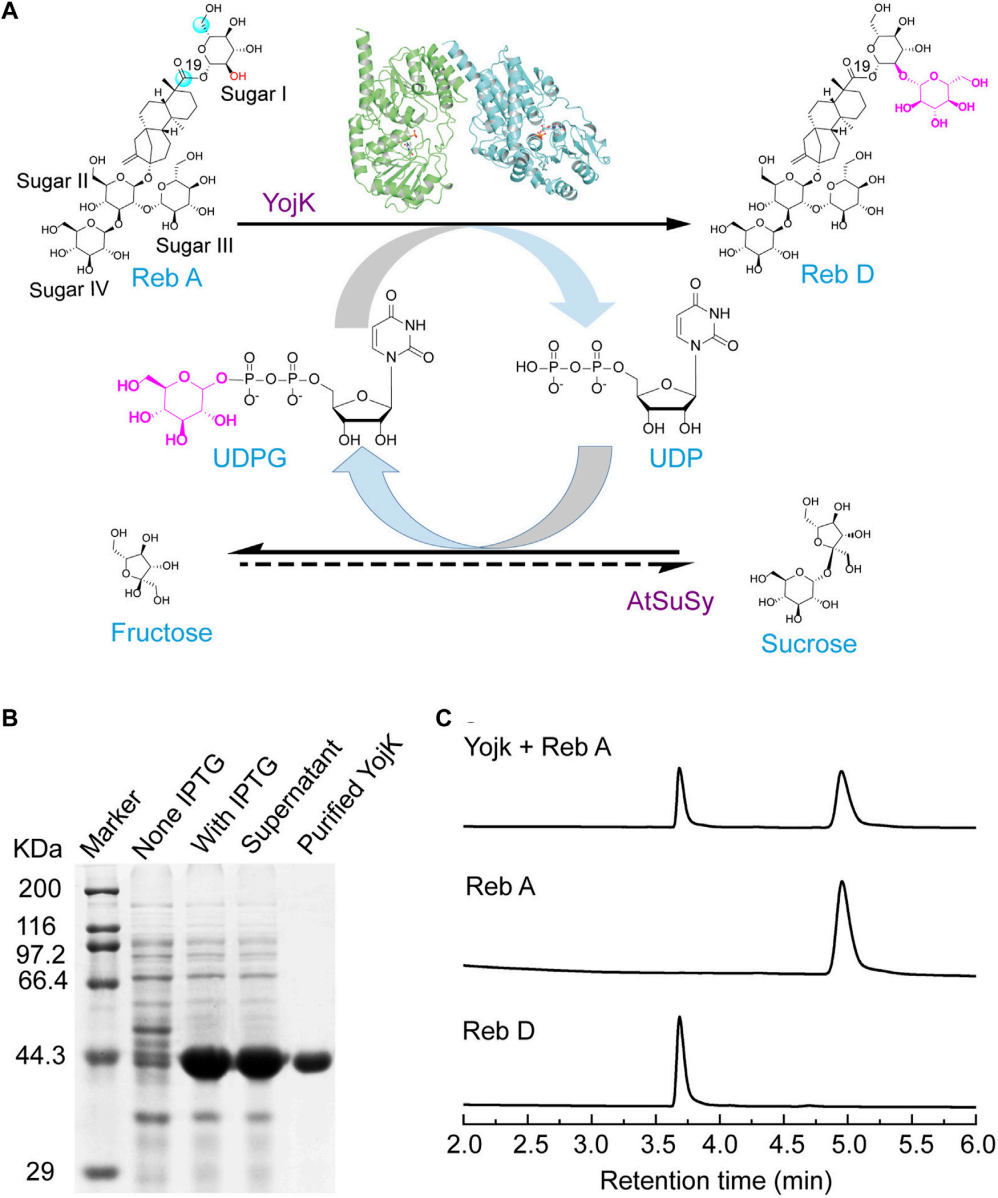
YojK has the catalytic ability to glycosylate Reb A to produce Reb D. **(A)** Scheme of the cascade reaction of YojK coupled with sucrose synthase *At*SuSy used for the recycle of UDPG. **(B)** SDS-PAGE analysis of the heterologous expression of YojK. **(C)** HPLC analysis of the glycosylation of Reb A to produce Reb D by YojK. Commercial Reb A and Reb D were used as standards.

To solve the solubility problem of plant-derived glycosyltransferases (Cai et al., 2017; Lemmerer et al., 2019; Chen et al., 2020; Shu et al., 2020), YojK, a Leloir O-glycosyltransferase from *B. subtilis* 168, was selected to determine whether it had the catalytic ability to glycosylate Reb A to form Reb D (Figure 1A), as it exhibits prominent glycosylation activity to various substrates like other bacterial glycosyltransferases with a large acceptor binding pocket (Strobel et al., 2013; Zhou et al., 2013; Pandey et al., 2014). Previous studies show that it can glycosylate substrates with large size like ginsenosides, crocins, flavonols and flavones (Luo et al., 2015; Pandey et al., 2019; Wang et al., 2019). At first, YojK could be recombinantly expressed in *E. coli* BL21 (DE3) and most of them were at soluble fraction (Figure 1B). Then, the purified YojK was used to determine the glycosylation of Reb A. To our delight, it was found that Reb D was well synthesized by YojK with UDPG as the glycosyl donor (Figure 1C), which was confirmed by LC-MS and NMR (Supplementary Figures S2–S9).

Next, enzyme kinetic parameters of YojK were determined by monitoring the formation of Reb D under the optimal temperature and pH conditions, which were 35°C and pH 8.0 (Supplementary Figure S10), respectively. YojK displays a *K*_m_ value of 210.52 ± 11.70 μM and a *k*_cat_ value of 1.08 ± 0.05 min^−1^ with Reb A as the substrate (Supplementary Figure S10), indicating that YojK has a lower catalytic activity towards Reb D than other reported glycosyltransferases (Chen et al., 2018; Wang et al., 2020). Therefore, it is necessary to perform structure-guided engineering to improve its catalytic activity for potential practical application after understanding its catalytic mechanism.

### Overall structure of YojK

**FIGURE 2:**
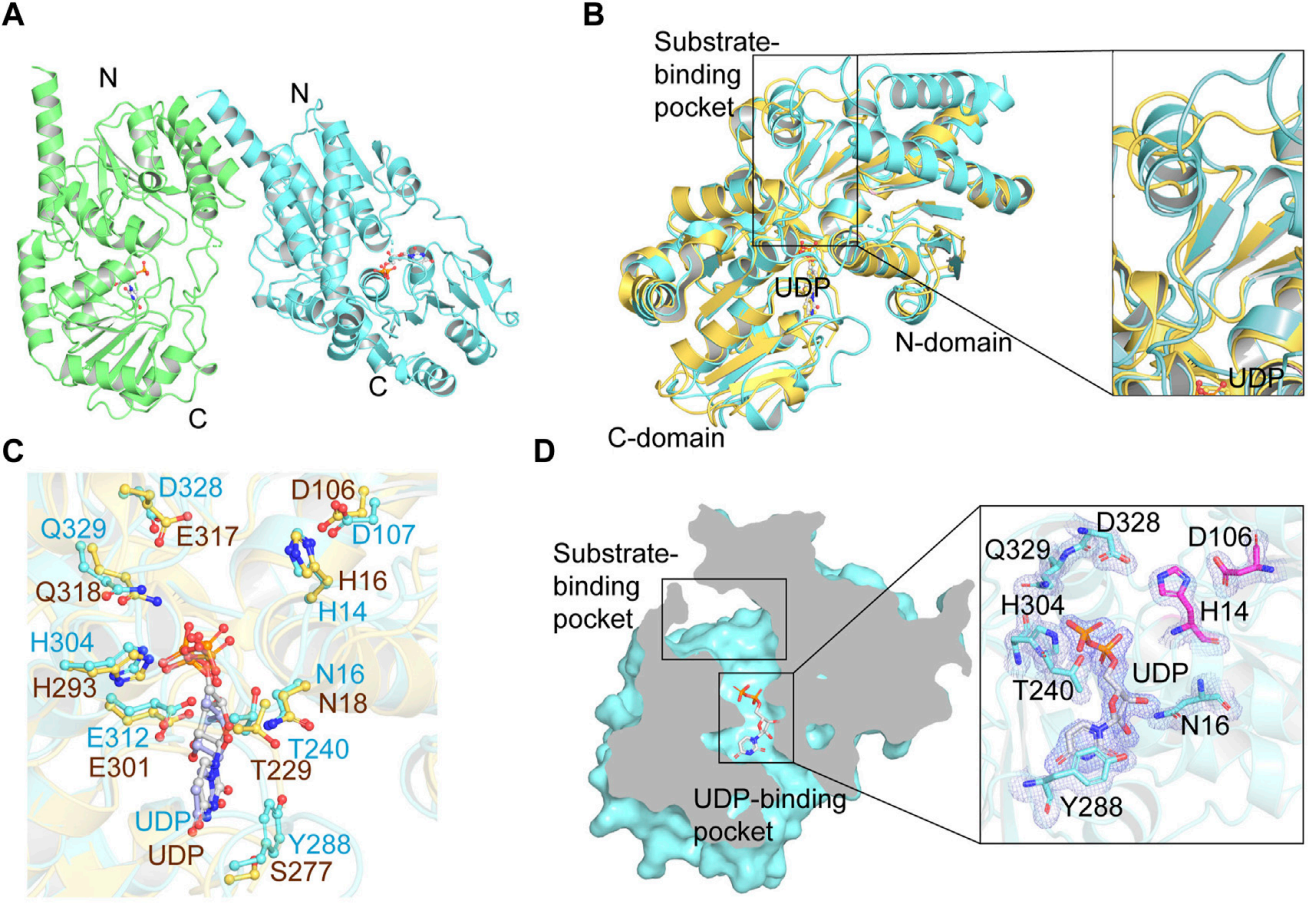
Crystal structure analysis of YojK-UDP complex. **(A)** Overall structure of YojK in complex with UDP. One asymmetric unit contains two monomers, which are colored in green and cyan, respectively. **(B)** Structural comparison of YojK and YjiC (PDB ID: 6kqx). The crystal structures of YojK and YjiC are colored in cyan and yellow, respectively. A detailed structure comparison of the substrate binding pocket is shown and marked with black rectangle. **(C)** Comparison of the UDP binding site in YojK and YjiC. YojK is colored in cyan while YjiC is colored in yellow. Atom oxygen and nitrogen are colored in red and blue, respectively. **(D)** Cross-section of YojK highlights the substrate-binding pocket and the UDP binding site. A detailed electron density map of the UDP-binding site and the active site (H14-D107 catalytic triad colored in purple) in YojK is displayed. The 2Fo–Fc map (light blue) of the UDP binding site is contoured at 1.0σ.

To better understand the molecular mechanism of YojK to catalyze the formation of Reb D from Reb A, we solved the crystal structure of YojK in complex with uridine diphosphate (UDP) and refined it to 1.90 Å solution (Figure 2A). The summary of data collection and refinement was shown in Supplementary Table S3. It consists of two monomers in each asymmetric unit. Similar to other bacterial glycosyltransferases, it folds a typical GT-B characteristic conformation containing two different β/α/β Rossmann domains connected by a variable loop region (V210-P224): a flexible N-domain (M1-F209) and a relatively rigid C-domain (F225-M405) (Figure 2B), which can be verified by superimposing the crystal structure of YojK with another bacterial glycosyltransferase YjiC (Figure 2B) (Liu et al., 2021). The flexible N-domain will allow YojK to accommodate various acceptor substrates, while the rigid C-domain contains a typical plant secondary product glycosyltransferase (PSPG) motif (Y288-Q329) with high conservation of sequence and structure (Supplementary Figure S11), which is responsible for recognizing and binding the sugar donor. Similar to other glycosyltransferases, the catalytic site (H14-D107 catalytic triad at YojK) locates at the cleft region and closes to the center of the sugar donor and the acceptor substrate, and most of UDPG binding sites (except Y288 and D328) are highly conserved (Figure 2C). However, the substrate binding pocket is highly plastic and flexible (Figures 2B,D), and a large substrate binding pocket is observed in the crystal structure of YojK (Figure 2D), indicating that it has an ability to accommodate diverse substrates, like the large substrate Reb A.

### Structure-guided engineering to improve the catalytic activity of YojK

**FIGURE 3:**
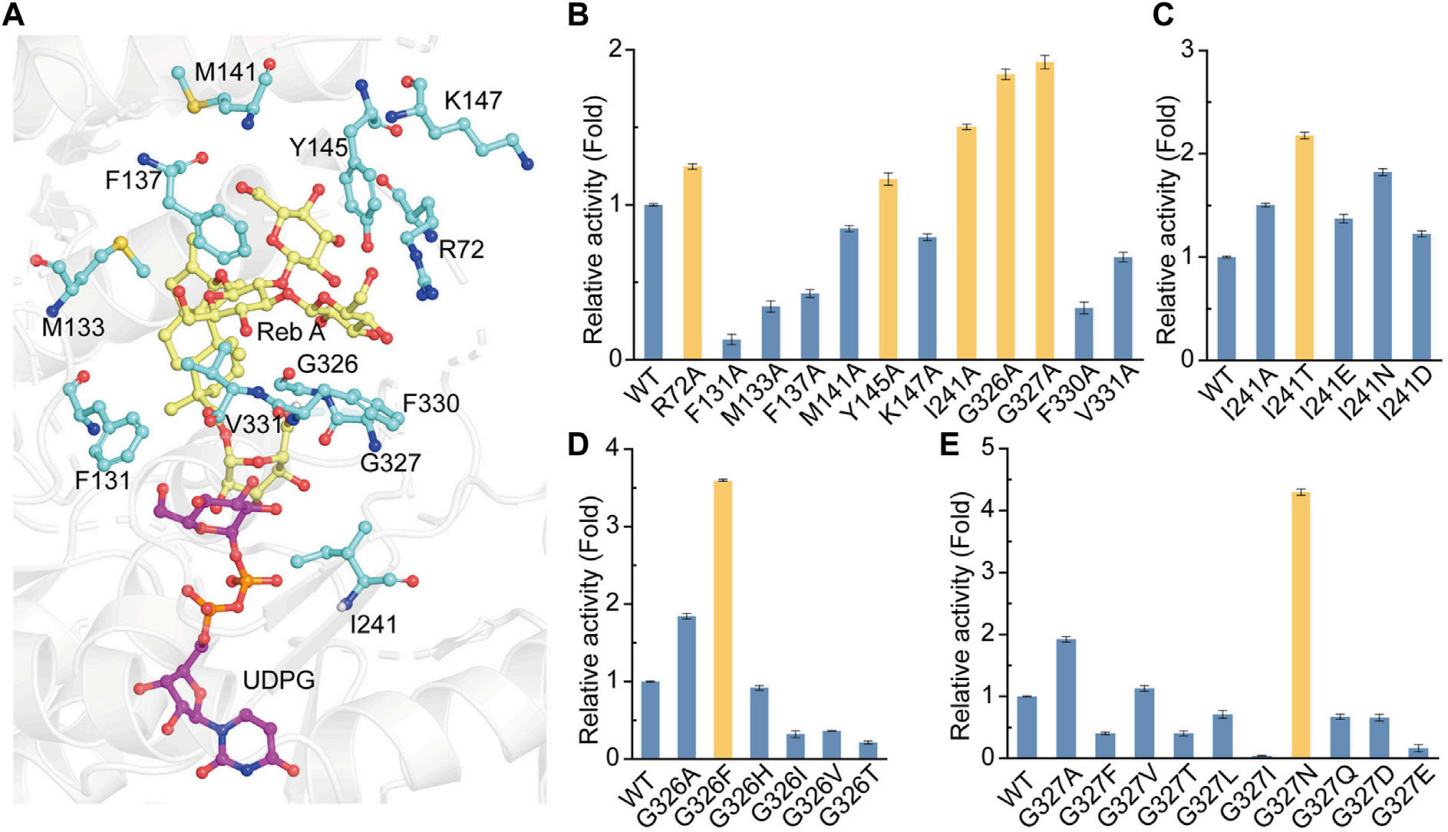
Site-directed mutagenesis of YojK to improve its catalytic activity. **(A)** Location of Reb A (yellow sticks), UDPG (purple sticks) and critical residues (cyan sticks) involved in binding are showed based on molecular docking. Atom oxygen and nitrogen are colored in red and blue, respectively. **(B)** Relative activities of alanine-scanning mutants of selected residues. The mutants with the improvement of the catalytic activity of YojK are highlighted in orange. **(C)** Relative catalytic activities of different I241 mutants. **(D)** Relative catalytic activities of different G326 mutants. **(E)** Relative catalytic activities of different G327 mutants. The catalytic activity of wild type (WT) YojK is normalized to one. The mutant with the highest catalytic activity is colored in orange. Error bars mean the standard deviation of three repeats.

**FIGURE 4:**
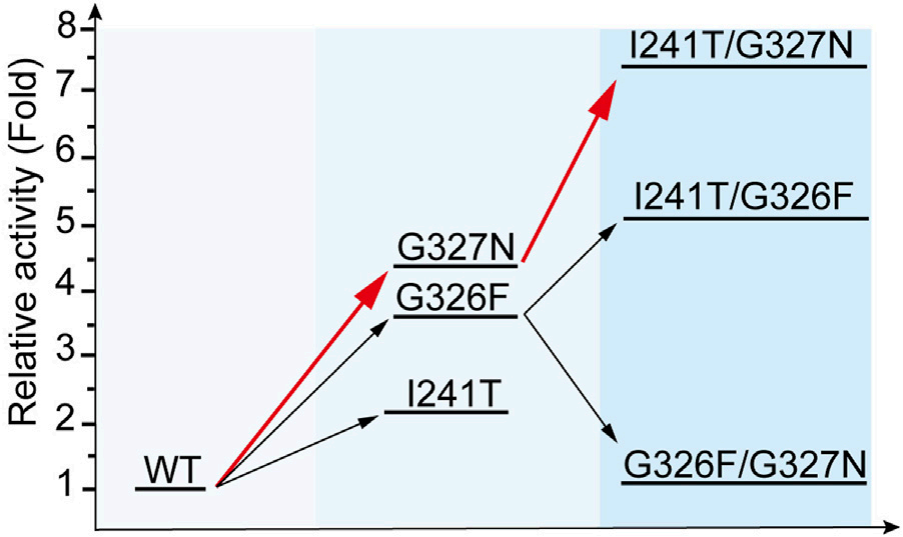
Iterative evolution of YojK. Stepwise improvement of catalytic activity of YojK towards Reb D. The catalytic activity of wild type YojK is normalized to one. The route with the highest relative catalytic activity of mutants by iterative evolution of YojK is presented in red.

With the crystal structure of YojK, we next performed structure-based engineering to improve its catalytic activity for the glycosylation of Reb A to produce Reb D. Probably due to low binding affinity between Reb A and YojK according to its enzymatic parameters (Supplementary Figure S10), the crystal structure of YojK in complex with Reb A and UDPG was not obtained. Thus, molecular docking was employed to dock Reb A and UDPG to the crystal structure of YojK-UDP complex using Discovery studio. It shows that R72, F131, M133, F137, M141, Y145, K147, I241, G326, G327, F330, and V331 are critical residues involved in binding and shaping the pocket (Figure 3A). As most of them locate at substrate access tunnel entrance of the enzyme, it suggests that these residues would tremendously determine the catalytic activity of YojK by affecting the migration of substrate (Cheng et al., 2021). Next, we performed alanine-scanning mutagenesis to analyze how these residues affect the catalytic activity of YojK to glycosylate Reb A to form Reb D (Figure 3B). It showed that I241A, G326A, and G327A obviously improved the catalytic activity of YojK with 1.51-fold, 1.84-fold, and 1.92-fold increase than that of wild type, respectively (Figure 3B). R72A and Y145A also increased the catalytic activity of YojK, but with a slight improvement. Other mutants (F131A, M133A, F137A, M141A, K147A, F330A, and V331A) negatively affected the catalytic activity of YojK. Consequently, I241, G326, and G327 were selected for further analysis to improve the catalytic activity of YojK. The I241T and I241N mutants could further improve the catalytic activity of YojK with a 2.18-fold and 1.82-fold increase, respectively, but not I241E or I241D mutant (Figure 3C). For residue G326, only G326F delivered a better catalytic activity than G326A, with 3.59-fold increase (Figure 3D). For residue G327, only G327N further improved the catalytic activity of YojK with 4.30-fold increase, but not other eight variant (Figure 3E). Then, these three mutants I241T, G326F, and G327N were used for combinatorial mutagenesis to further improve catalytic activity of YojK. It showed that variants YojK-I241T/G326F and YojK-I241T/G327N could further improve their catalytic activity compared with each single mutant of them, with 5.24-fold and 7.35-fold increase, respectively (Figure 4), but not YojK-G326F/G327N. This variant only keeps the similar catalytic activity as wild-type YojK, without additive effect from G326F and G327N. Next, enzymatic parameters of all these mutants were analyzed to understand how they potentially affect the catalytic activity of YojK (Table 1). Based on the corresponding value of *k*_cat_, *K*_m_, and *k*_cat_/*K*_m_, both of I241T and G327N not only increase the bind affinity between Reb A and YojK, but also improve the catalytic ability of YojK towards Reb A, while G326F decreases the binding affinity between Reb A and YojK even with a dramatic increase of its catalytic ability. This could explain why the variant YojK-I241T/G327N has a better catalytic efficiency than YojK-I241T/G326F. Thus, the variant YojK-I241T/G327N was selected to further analyze its catalytic mechanism and optimize the reaction conditions to perform a large-scale preparation of Reb D.

**TABLE 1:** Kinetic parameters of YojK and its variants towards Reb D.

Variants	*K*_m_ (μM)	*k*_cat_ (min^−1^)	*k*_cat_/*K*_m_ (mM^−1^ min^−1^)
WT	210.52 ± 11.70	1.08 ± 0.05	5.13 ± 0.09
I241T	197.74 ± 9.25	1.34 ± 0.07	6.78 ± 0.07
G326F	290.16 ± 8.34	9.21 ± 0.09	31.70 ± 0.75
G327N	176.23 ± 10.62	5.02 ± 0.07	28.49 ± 0.53
I241T/G326F	347.63 ± 24.23	15.45 ± 0.03	44.45 ± 2.25
I241T/G327N	193.07 ± 7.30	10.76 ± 0.05	55.75 ± 1.34

### Molecular dynamics simulations of the variant YojK-I241T/G327N

**FIGURE 5:**
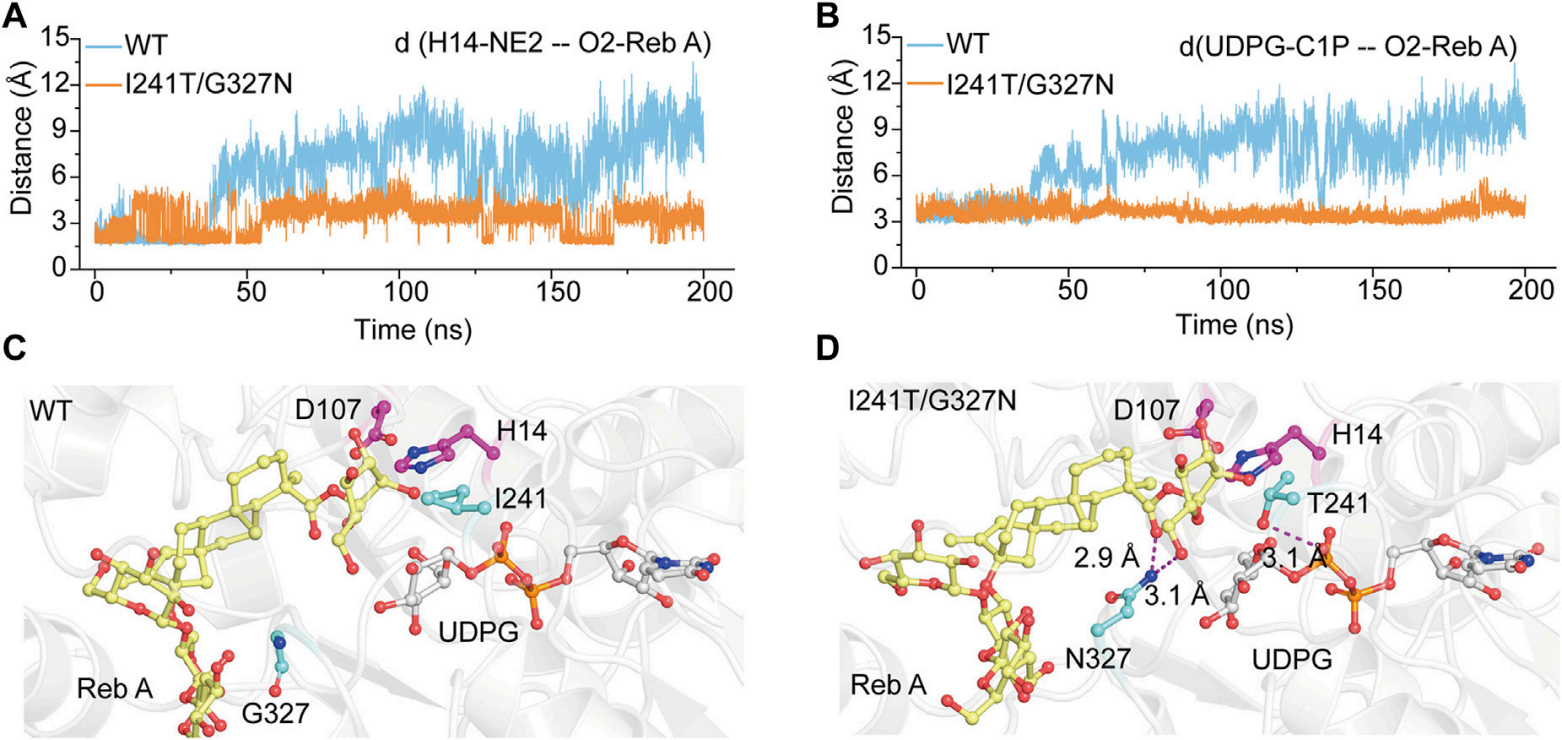
The representative catalytic conformations of WT-UDPG-Reb A and YojK-I241T/G327N-UDPG-Reb A in MD stimulations. **(A)** The distance between the atom O2 of the acceptor glucose of Reb A and the atom NE2 of the catalytic residue H14 along 200 ns MD simulations. **(B)** The distance between the atom O2 of the acceptor glucose of Reb A and the atom C1P of UDPG along 200 ns MD simulations. **(C)** The representative catalytic conformation of YojK in MD simulations. Residues I241 and G327 are shown as cyan sticks. **(D)** The representative catalytic conformation of YojK-I241T/G327N in MD simulations. The new hydrogen bonds are indicated in the purple dash lines. The distances are shown. Residues T241 and N327 are shown as cyan sticks. The active sites (H14-D107) are shown as purple sticks. Reb A and UDPG are displayed as yellow and white sticks, respectively. Atom oxygen and nitrogen are colored in red and blue, respectively.

To elucidate the catalytic mechanism of the variant YojK-I241T/G327N with the improvement of the catalytic activity toward Reb D, unconstrained MD simulations (200 ns) of the complexes WT-UDPG-Reb A and YojK-I241T/G327N-UDPG-Reb A were executed after molecular docking. Based on the catalytic mechanism of UDP-glycosyltransferases in the catalytic process, catalytic residue H14 of YojK firstly takes a proton from the hydroxyl group of the glucosyl unit at the C19-carboxylate position of Reb A to generate nucleophile (Figure 1A), which then adopts S_N_2-like mechanism to attack the C1 carbon of the glucose of the sugar donor UDPG to form the β-1,2-linkage (Figure 1A) (Rahimi et al., 2019). Thus, the distance between the atom O2 of the hydroxyl group of Reb A and the NE2 of the catalytic residue H14, and the distance between the atom O2 of the hydroxyl group of Reb A and the C1P of UDPG along 200 ns MD simulations were used to analyze the catalytic efficiency of wild type YojK and variant YojK-I241T/G327N. As shown in Figure 5A, the distance between the atom O2 of Reb A and the NE2 of H14 in mutant YojK-I241T/G327N kept in the hydrogen bond range (around 3.6 Å) along 200 ns MD simulations, while this distance became out of the hydrogen bond range after 35 ns for WT-UDPG-Reb A. Meanwhile, the distance between the atom O2 of the hydroxyl group of Reb A and the C1P of UDPG exhibited similar tendency for YojK and variant YojK-I241T/G327N (Figure 5B). Therefore, the representative catalytic conformations of YojK-I241T/G327N-UDPG-Reb A complex and WT-UDPG-Reb A complex were then studied to analyze the conformational changes (Figures 5C,D). It was found that N327 formed new strong hydrogen bonding interactions with the hydroxyl group at the hydroxyl group at C6 position of sugar I of Reb A and the C19-carboxylate position of Reb A in the typical conformation of YojK-I241T/G327N-UDPG-Reb A complex (Figures 1A, 5D). Moreover, residue T241 formed additional hydrogen bond with phosphate group of UDPG (Figure 5D), which could stabilize the sugar donor UDPG during glycosylation. Therefore, it suggests that the formation of new hydrogen bonding interactions accounts for the improvement of catalytic efficiency of YojK-I241T/G327N variant.

### Optimization and preparation of Reb D in cascade reactions

**FIGURE 6:**
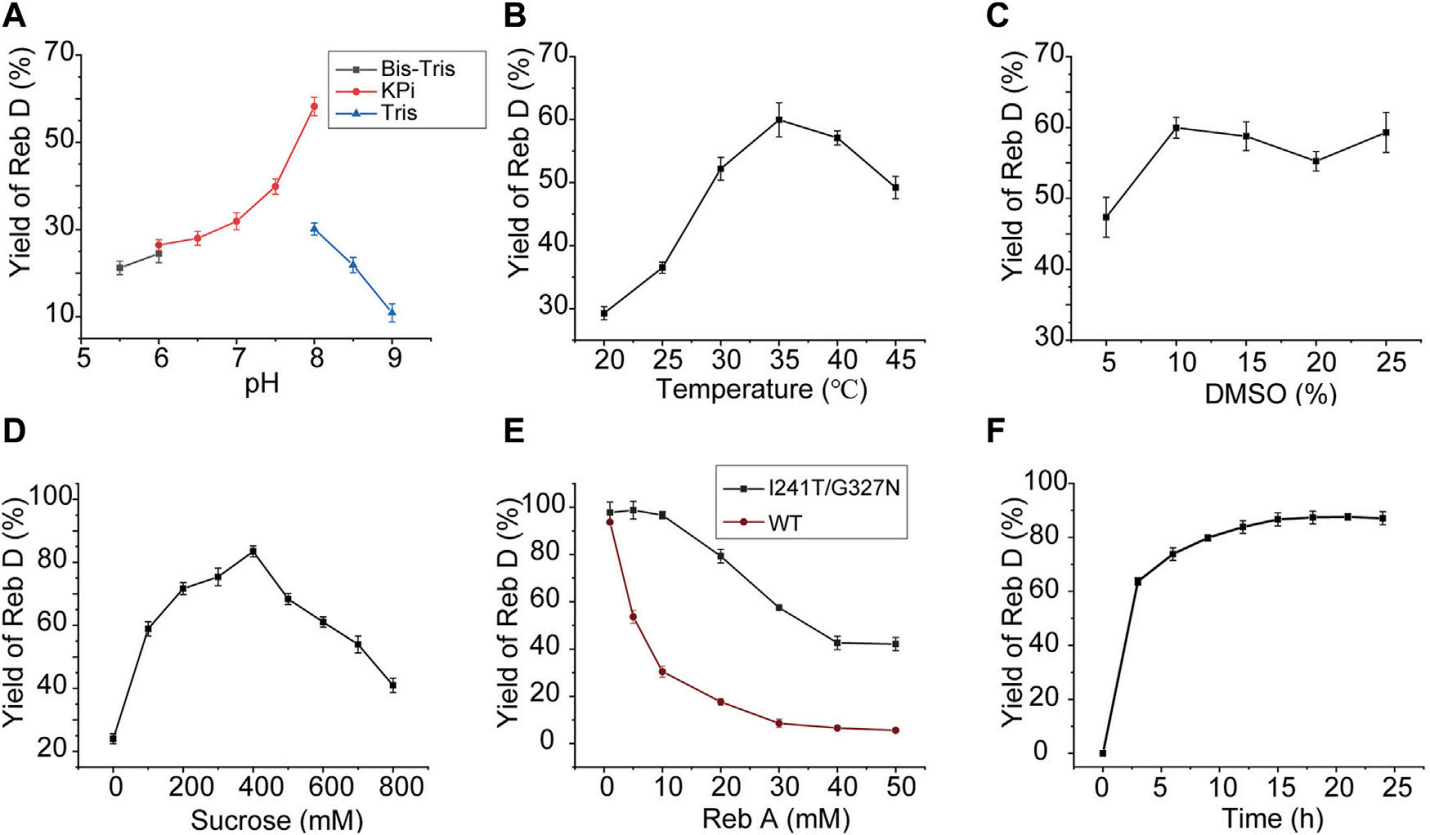
Optimization of the cascade reaction conditions for YojK-I241T/G327N-*At*SuSy. **(A)** Optimization of pH. **(B)** Optimization of reaction temperature. **(C)** Optimization of the ratio of DMSO. **(D)** Optimization of sucrose concentration. **(E)** Comparison of the synthetic efficiency of Reb D at different concentrations of Reb A for YojK-I241T/G327N-*At*SuSy and YojK-*At*SuSy cascade reaction systems. **(F)** Optimization of the reaction time on a large-scale preparation of Reb D. Error bars mean the standard deviation of three repeats.

To synthesize Reb D on a scale preparation with cost-effective UDPG, sucrose synthase *At*SuSy from *A. thaliana* was employed to recycle UDPG from low-cost sucrose and UDP (Figure 1A) (Schmolzer et al., 2016; Wang et al., 2016), and the cascade reaction of YojK-I241T/G327N-*At*SuSy should be optimized. At first, the co-expression of YojK-I241T/G327N and *At*SuSy in *E. coli* BL21 (DE3) was analyzed. It showed that they could be recombinantly co-expressed in *E. coli* BL21 (DE3) and most of them were at the soluble fraction (Supplementary Figure S12). Next, the cascade reaction conditions were optimized using cell lysates of this co-expression system. To balance the optimal pH between YojK-I241T/G327N variant and *At*SuSy, the pH of reaction buffer was re-analyzed. It showed that pH 8.0 in potassium phosphate (KPi) buffer delivered the best catalytic activity for the synthesis of Reb D, but not Tris-HCl buffer (Figure 6A). The effects of temperature, reaction time, DMSO ratio and the concentration of sucrose were also investigated with the optimal pH. The reaction temperature at 35°C, the ratio of co-solvent DMSO at 10% and the concentration of sucrose at 400 mM gave the maximum yield of Reb D at 83.47% (Figures 6B–D). More sucrose even impaired the bioconversion of Reb A to Reb D (Figure 6D). Since divalent cations, such as Mn^2+^ and Mg^2+^, inhibit the catalytic activity of sucrose synthase in both of cleavage and synthesis direction, and lead to the decomposition of nucleoside diphosphate glucose (NDP-glucose) (Diricks et al., 2015; Schmolzer et al., 2016), the effect of the addition of metal ions was not investigated in this cascade reaction. Last, the effect of the concentration of Reb A was then determined after optimizing the above parameters. For the YojK-I241T/G327N-*At*SuSy cascade reaction, the yield of Reb D was decreased from 97.78% to 42.12%, when the concentration of Reb A was gradually increased from 1 to 50 mM (Figure 6E). To our delight, the production of Reb D still kept at high conversion yield when the concentration of Reb A was lower than 20 mM (Figure 6E). In contrast, wild type YojK-*At*SuSy cascade reaction displayed a low yield of Reb D even at low concentration of Reb A less than 10 mM (Figure 6E), which might be ascribed to low activity of wild type YojK.

Last, a large-scale preparation of Reb D was carried out with the above optimized conditions. To avoid the shortage of UDPG, additional 1 mM UDPG was added to 20 ml YojK-*At*SuSy cascade reaction system. It was found that 20.59 g/L Reb D with an excellent yield of 91.29% was synthesized from 19.32 g/L (20 mM) Reb A after 15 h (Figure 6F), which is better than the previous studies (Chen et al., 2020). Therefore, together with the result by circumventing soluble expression problem of plant-based glycosyltransferases, it suggests that this YojK-I241T/G327N-*At*SuSy cascade reaction system after structure-guided engineering of YojK provides the feasibility for industrial scale-production of Reb D.

## Conclusion

In summary, a novel bacterial glycosyltransferase YojK from *B. subtilis* 168 [recognized as Generally Recognized as Safe (GRAS) strain] with the catalytic ability to glycosylate Reb A to synthesize Reb D, a plant-derived zero-calorie sweetener with high-intensity sweetness and a much less lingering bitter aftertaste, was identified and characterized. Unlike most of plant-based glycosyltransferases, it could be recombinantly expressed in *E. coli* BL21 (DE3) with high solubility. We then solved the crystal structure of glycosyltransferase YojK to improve its catalytic activity by structure-guided engineering. A double mutant YojK-I241T/G327N was obtained, which allowed to prepare Reb D on a large-scale with an excellent yield of 91.29% with the recycle of UDPG catalyzed by sucrose synthase *At*SuSy. Therefore, this study provides a novel glycosyltransferase YojK-I241T/G327N to set up an efficient enzymatic cascade reaction with sucrose synthase *At*SuSy for potential industrial scale-production of Reb D in an economically effective manner.

## Data Availability

The original contributions presented in the study are included in the article/Supplementary Material, further inquiries can be directed to the corresponding author.
