# Supplementary material for: Highly efficient production of rebaudioside D enabled by structure-guided engineering of bacterial glycosyltransferase YojK
Source: Front Bioeng Biotechnol. 2022 Aug 25;10:985826. doi: 10.3389/fbioe.2022.985826 (PMC9452701; doi:10.3389/fbioe.2022.985826)
Supplement: Supplementary file 1 [file DataSheet1.docx]

Supplementary Material

# Supplementary Figures and Tables

## Supplementary Tables

**Supplementary Table 1 Strains and plasmids used in this study**

| Strains or Plasmids | Description | Sources |
| --- | --- | --- |
| Strains |  |  |
| Top10 | wild type | Novagen |
| BL21(DE3) | wild type | Novagen |
| BL21-*YojK* | *E. coli* BL21(DE3) harboring plasmid pET-21b(+)-*YojK* | this study |
| BL21-*YojK*(mutants) | *E. coli* BL21(DE3) harboring plasmid pET-21b(+)-*YojK(*mutants) | this study |
| BL21-YojK-*At*SuSy | *E. coli* BL21(DE3) harboring plasmid pACYCDuet-1-YojK-AtSuSy | this study |
| BL21-YojK-I241T/G327N-*At*SuSy | *E. coli* BL21(DE3) harboring plasmid pACYCDuet-1-*YojK*-I241T/G327N-*AtSuSy* | this study |
| Plasmids |  |  |
| pET21b-*YojK* | pET-21b(+) carry a glycosyltransferase YojK gene from *B. subtilis* 168, Amp^R^ | this study |
| pET21b-*YojK*(mutants) | pET-21b(+) carry a glycosyltransferase YojK mutant gene, Amp^R^ | this study |
| pACYCDuet-1-*YojK*-*AtSuSy* | pACYCDuet-1 carry a glycosyltransferase YojK and a sucrose synthase *At*SuSy gene, Cm^R^ | this study |
| pACYCDuet-1-*YojK*-I241T/G327N-*AtSuSy* | pACYCDuet-1 carry a glycosyltransferase YojK mutant YojK-I241T/G327N and a sucrose synthase *At*SuSy gene, Cm^R^ | this study |

**Supplementary Table 2 Primers for YojK mutagenesis used in this study**

| Primer name^a^ | Sequence (5' to 3') |
| --- | --- |
| R72A-F | ATGAAGAAGCTGATTTCACAGAAATGCTCTGCGC |
| R72A-R | TGAAATCAGCTTCTTCATCGCCGGTTGCG |
| F131A-F | GTACAACCGCTGCGATGAATGAGGAATTTGCGAAG |
| F131A-R | TCATCGCAGCGGTTGTACACAATGAAAATCTTGGCAGC |
| M133A-F | CCTTTGCGGCTAATGAGGAATTTGCGAAGGAAATGATGG |
| M133A-R | CCTCATTAGCCGCAAAGGTTGTACACAATGAAAATCT |
| F137A-F | ATGAGGAAGCTGCGAAGGAAATGATGGGAGCGTA |
| F137A-R | CCTTCGCAGCTTCCTCATTCATCGCAAAGGTTGTACAC |
| M141A-F | CGAAGGAAGCTATGGGAGCGTACATGAAAGGATC |
| M141A-R | CTCCCATAGCTTCCTTCGCAAATTCCTCATTCATCG |
| Y145A-F | TGGGAGCGGCTATGAAAGGATCACTTGAAGATTCGCC |
| Y145A-R | CTTTCATAGCCGCTCCCATCATTTCCTTCGCAA |
| K147A-F | GCGTACATGGCTGGATCACTTGAAGATTCGCCTCAT |
| K147A-R | GTGATCCAGCCATGTACGCTCCCATCATTTCCTT |
| I241A-F | GGGAACCGCTTTTAATAATCAAAAGCAGTTTTTTAATCAATGCCTTGAAGTG |
| I241A-F | TTATTAAAAGCGGTTCCCATTGAAATAAACAGCACGTTTTCA |
| G326A-F | TTCCGATGGCTGGCGACCAATTTGTTGTCGCA |
| G326A-R | GGTCGCCAGCCATCGGAATGACAACGAGCGG |
| G327A-F | CGATGGGAGCTGACCAATTTGTTGTCGCAGATCAGGTA |
| G327A-R | ATTGGTCAGCTCCCATCGGAATGACAACGAGC |
| F330A-F | GCGACCAAGCTGTTGTCGCAGATCAGGTAGAAAAAGTC |
| F330A-R | CGACAACAGCTTGGTCGCCTCCCATCGGAA |
| I241T-F | TTCAATGGGAACCACTTTTAATAATCAAAAGC |
| I241T-R | GCTTTTGATTATTAAAAGTGGTTCCCATTGAA |
| I241E-F | TTCAATGGGAACCGAATTTAATAATCAAAAGC |
| I241E-R | GCTTTTGATTATTAAATTCGGTTCCCATTGAA |
| I241N-F | TGGGAACCAATTTTAATAATCAAAAGCAGTTTTTTAATCAATGCCTTGAAG |
| I241N-R | TATTAAAATTGGTTCCCATTGAAATAAACAGCACG |
| I241D-F | TGGGAACCGATTTTAATAATCAAAAGCAGTTTTTTAATCAATGCCTTGAAG |
| I241D-R | TATTAAAATCGGTTCCCATTGAAATAAACAGCACG |
| G326F-F | TTCCGATGTTTGGCGACCAATTTGTTGTCGCA |
| G326F-R | GGTCGCCAAACATCGGAATGACAACGAGCGG |
| G326H-F | TTCCGATGCATGGCGACCAATTTGTTGTCGCA |
| G326H-R | GGTCGCCATGCATCGGAATGACAACGAGCGG |
| G326I-F | TTCCGATGATTGGCGACCAATTTGTTGTCGCA |
| G326I-R | GGTCGCCAATCATCGGAATGACAACGAGCGG |
| G326V-F | TTCCGATGGTTGGCGACCAATTTGTTGTCGCA |
| G326V-R | GGTCGCCAACCATCGGAATGACAACGAGCGG |
| G326T-F | TTCCGATGCTTGGCGACCAATTTGTTGTCGCA |
| G326T-R | GGTCGCCAAGCATCGGAATGACAACGAGCGG |
| G327F-F | CGATGGGATTTGACCAATTTGTTGTCGCAGATCAGGTA |
| G327F-R | ATTGGTCAAATCCCATCGGAATGACAACGAGC |
| G327V-F | CGATGGGAGTTGACCAATTTGTTGTCGCAGATCAGG |
| G327V-R | ATTGGTCAACTCCCATCGGAATGACAACGAGC |
| G327T-F | CGATGGGAACAGACCAATTTGTTGTCGCAGATCAGG |
| G327T-R | ATTGGTCTGTTCCCATCGGAATGACAACGAGC |
| G327L-F | CGATGGGACTTGACCAATTTGTTGTCGCAGATCAGG |
| G327L-R | ATTGGTCAAGTCCCATCGGAATGACAACGAGC |
| G327I-F | CGATGGGAATTGACCAATTTGTTGTCGCAGATCAGG |
| G327I-R | ATTGGTCAATTCCCATCGGAATGACAACGAGC |
| G327N-F | CGATGGGAAATGACCAATTTGTTGTCGCAGATCAGGTA |
| G327N-R | ATTGGTCATTTCCCATCGGAATGACAACGAGC |
| G327Q-F | CGATGGGACAAGACCAATTTGTTGTCGCAGATCAGGTA |
| G327Q-R | ATTGGTCTTGTCCCATCGGAATGACAACGAGC |
| G327D-F | CGATGGGAGATGACCAATTTGTTGTCGCAGATCAGGTA |
| G327D-R | ATTGGTCATCTCCCATCGGAATGACAACGAGC |
| G327E-F | CGATGGGAGAAGACCAATTTGTTGTCGCAGATCAGGTA |
| G327E-R | ATTGGTCTTCTCCCATCGGAATGACAACGAGC |
| G326F/G327N-F | CGATGTTTAATGACCAATTTGTTGTCGCAGATCAGGTA |
| G326F/G327N-F | ATTGGTCATTAAACATCGGAATGACAACGAGC |

a: A: alanine (Ala); R: Arginine (Arg); H: histidine (His); G: glycine (Gly); K: lysine (Lys); V: Valine (Val); I: isoleucine (Ile); F: phenylalanine (Phe); D: Aspartic acid (Asp); N: Asparagine (Asn); L: leucine (Leu); M: methionine (Met); Y: tyrosin (Tyr); T: threonine (Thr); E: glutamic acid (Glu); Q: glutamine (Gln).

**Supplementary** **Table 3 Data collection and refinement statistics of YojK crystals**

| Parameters | YojK |
| --- | --- |
| PDB code | 7VM0 |
| Data collection |  |
| Space group | P 1 2_1_ 1 |
| Cell dimensions |  |
| a, b, c (Å) | 46.60, 81.05, 100.68 |
| α, β, γ (⸰) | 90.00, 91.87, 90.00 |
| Resolution (Å) | 26.38 – 1.90 (1.97-1.90) |
| R_merge_ | 0.092 |
| R_pim_ | 0.035 |
| *I/σI* | 20.49 (3.68) |
| Completeness (%) | 98.35 (94.89) |
| Redundancy | 6.9 |
| Refinement |  |
| No. reflections | 57974 (5547) |
| R_work_/R_free_ | 0.2022/0.2408 |
| No. atoms | 6538 |
| B factors (Å ^2^ ) | 24.72 |
| R.m.s.deviations |  |
| bond lengths (Å) | 0.015 |
| bond angles (˚) | 1.94 |
| Ramachandran outliers (%) | 0.00 |
| Ramachandran favored (%) | 96.79 |

Note: values in parentheses are for the highest-resolution shell.

## Supplementary Figures


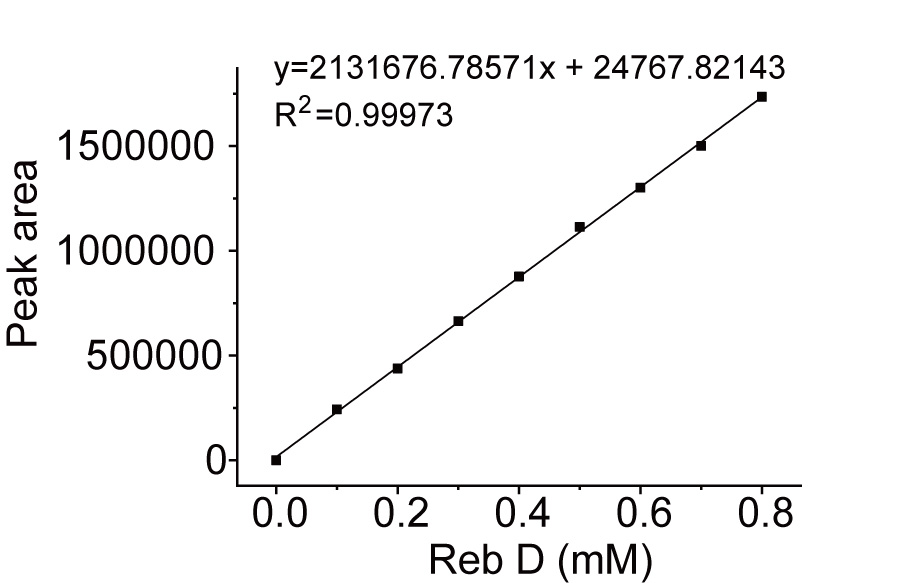


**Supplementary Figure 1.** Standard curve of Reb D.


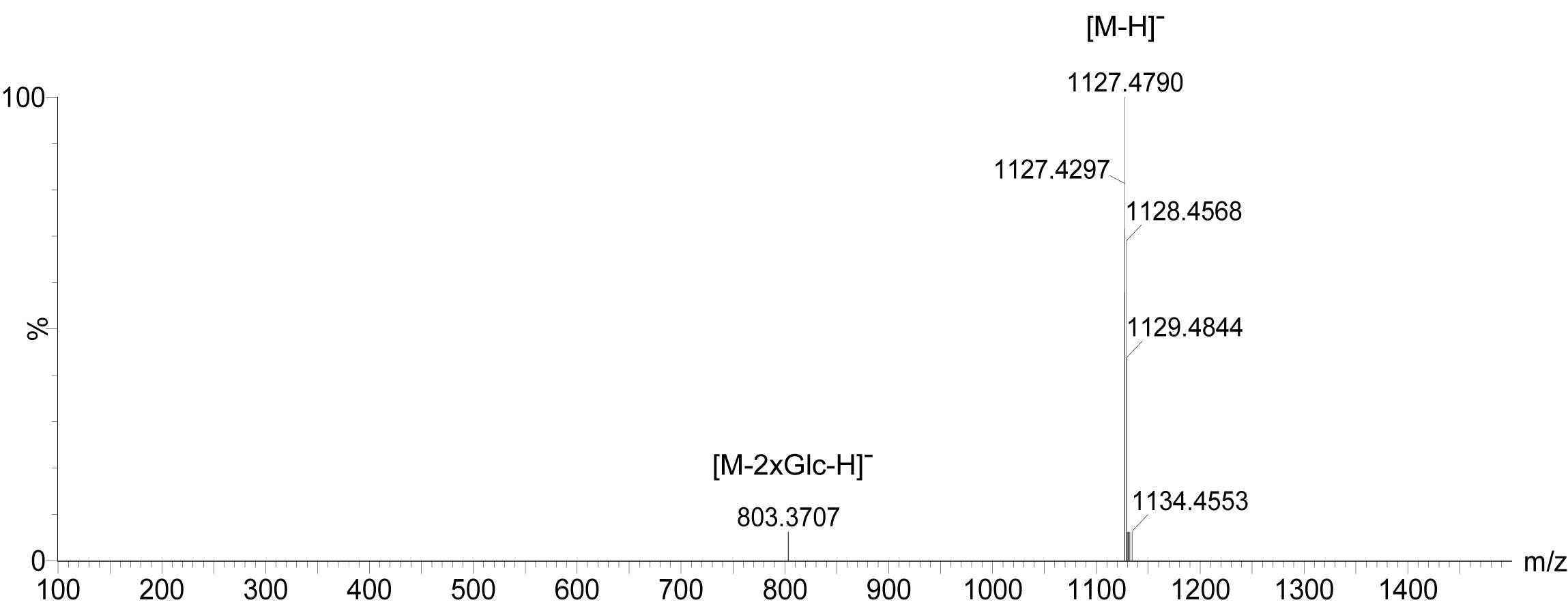


**Supplementary Figure 2** LC-MS analysis of the new product after glycosylating Reb A by YojK. HRMS (ESI^-^) calculated for C_50_H_80_O_28_ [M-H]^-^: 1127.4758; Found: 1127.4790.


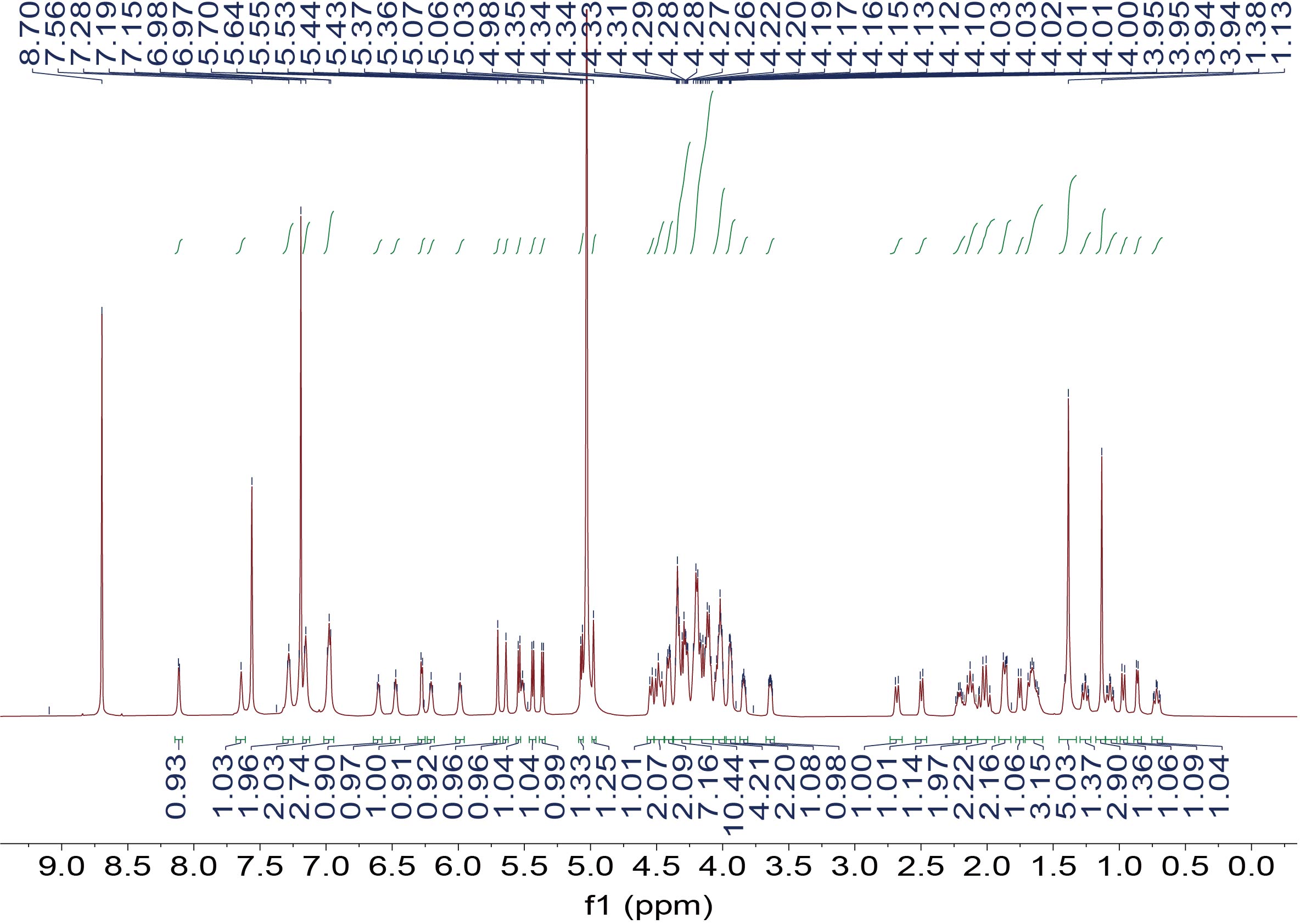


**Supplementary Figure 3** The ^1^H NMR spectrum of Reb D in Pyridine-*d*_5_ (600 MHz). **^1^H NMR** (600 MHz, Pyridine-*d_5_*) δ 8.11 (d, *J* = 4.5 Hz, 1H), 7.64 (s, 1H), 7.28 (t, *J* = 5.5 Hz, 2H), 7.16 (d, *J* = 6.1 Hz, 2H), 6.98 (t, *J* = 7.4 Hz, 3H), 6.60 (t, *J* = 5.3 Hz, 1H), 6.47 (t, *J* = 6.0 Hz, 1H), 6.28 (d, *J* = 6.4 Hz, 1H), 6.21 (t, *J* = 6.4 Hz, 1H), 5.99 (t, *J* = 5.8 Hz, 1H), 5.70 (s, 1H), 5.64 (s, 1H), 5.54 (d, *J* = 7.9 Hz, 1H), 5.44 (d, *J* = 7.9 Hz, 1H), 5.36 (d, *J* = 7.8 Hz, 1H), 5.07 (d, *J* = 7.5 Hz, 1H), 4.98 (s, 1H), 4.54 (d, *J* = 11.2 Hz, 1H), 4.48 (t, *J* = 14.2 Hz, 2H), 4.44 – 4.38 (m, 2H), 4.37 – 4.25 (m, 7H), 4.24 – 4.07 (m, 11H), 4.07 – 3.99 (m, 4H), 3.94 (ddd, *J* = 9.8, 4.8, 2.5 Hz, 2H), 3.84 (ddd, *J* = 9.0, 5.5, 3.0 Hz, 1H), 3.67 – 3.61 (m, 1H), 2.68 (d, *J* = 13.0 Hz, 1H), 2.50 (d, *J* = 11.2 Hz, 1H), 2.21 (td, *J* = 12.1, 6.7 Hz, 1H), 2.13 (t, *J* = 12.9 Hz, 2H), 2.02 (q, *J* = 17.1, 16.7 Hz, 2H), 1.91 – 1.82 (m, 2H), 1.75 (d, *J* = 11.3 Hz, 1H), 1.65 (ddd, *J* = 23.2, 16.1, 9.2 Hz, 3H), 1.38 (s, 5H), 1.26 (td, *J* = 13.1, 3.5 Hz, 1H), 1.13 (s, 3H), 1.07 (td, *J* = 13.5, 4.3 Hz, 1H), 0.97 (d, *J* = 12.3 Hz, 1H), 0.86 (d, *J* = 7.5 Hz, 1H), 0.72 (td, *J* = 13.4, 4.2 Hz, 1H) ppm.


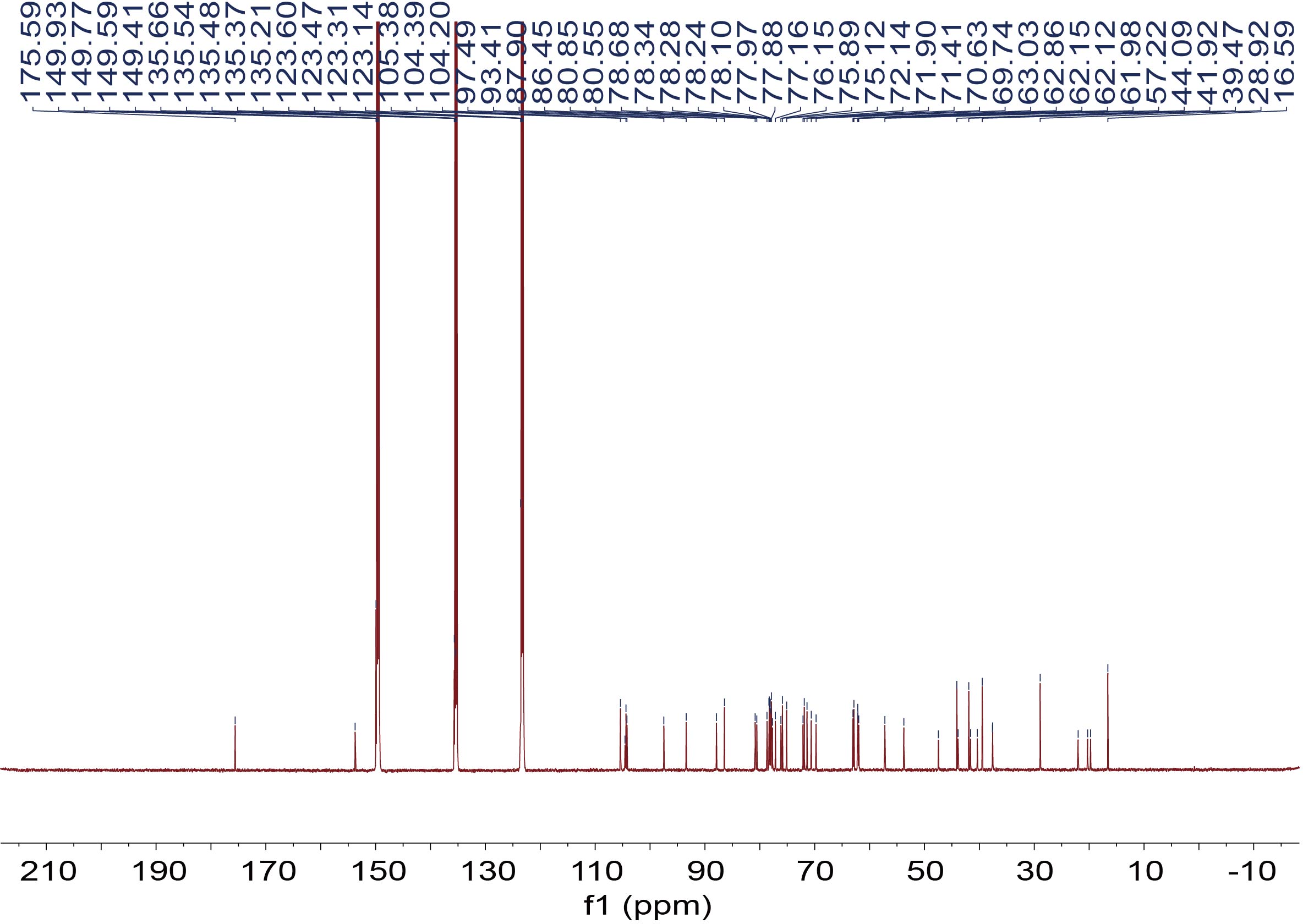


**Supplementary Figure 4** The ^13^C NMR spectrum of Reb D in Pyridine-*d*_5_ (151 MHz). **^13^C NMR** (151 MHz, Pyridine-*d*_5_) δ 175.6, 153.7, 149.9, 149.8, 149.6, 149.4, 135.7, 135.5, 135.5, 135.4, 135.2, 123.6, 123.5, 123.3, 123.1, 105.4, 104.6, 104.4, 104.2, 97.5, 93.4, 87.9, 86.5, 80.9, 80.6, 78.7, 78.3, 78.3, 78.2, 78.10, 78.0, 77.9, 77.7, 77.2, 76.2, 75.9, 75.1, 72.1, 71.9, 71.4, 70.6, 69.7, 63.0, 62.9, 62.2, 62.1, 62.0, 57.2, 53.8, 47.5, 44.1, 43.9, 41.9, 41.6, 40.4, 39.5, 37.6, 37.6, 28.9, 22.0, 20.3, 19.7, 16.6 ppm.


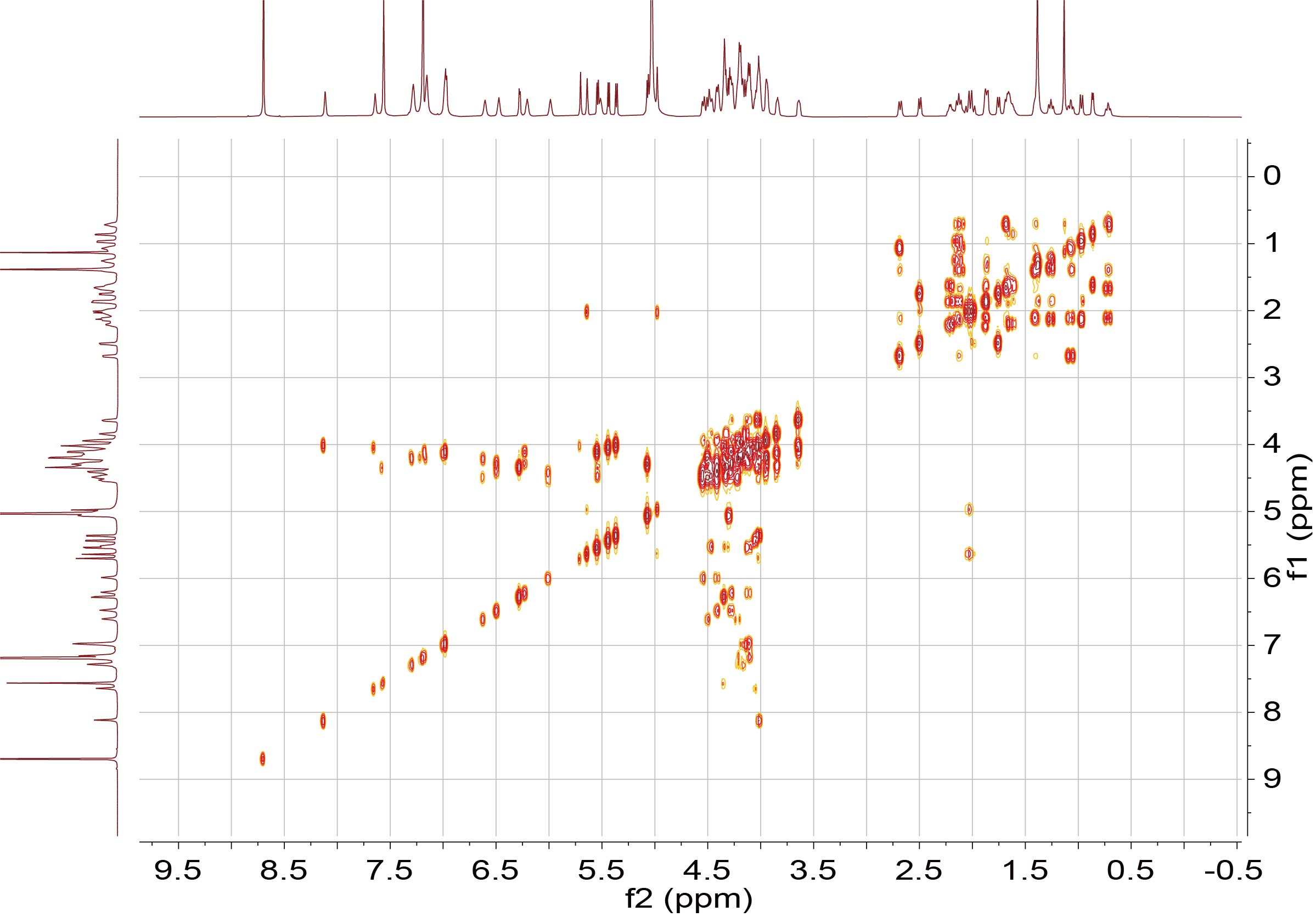


**Supplementary Figure 5** COSY of Reb D.


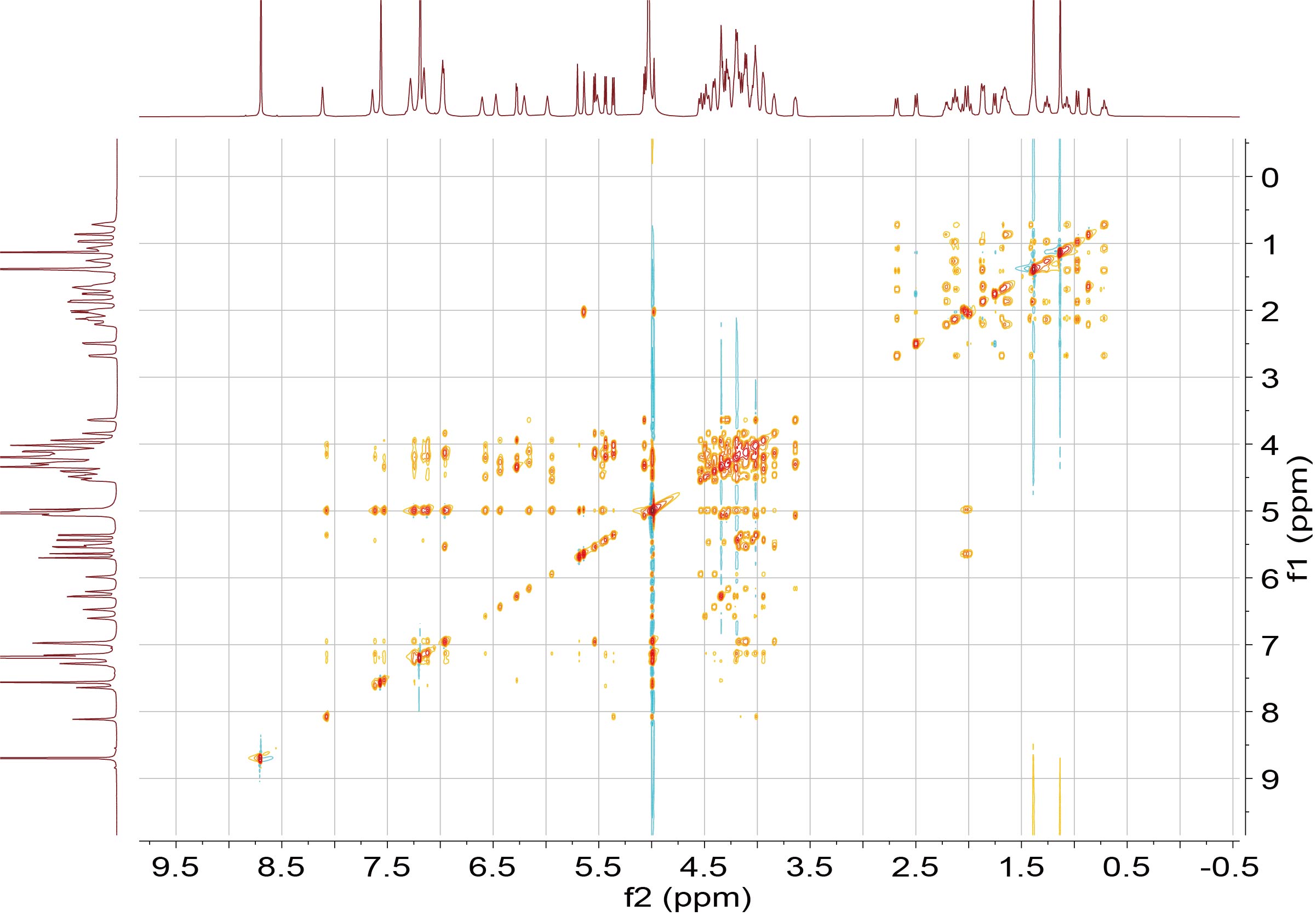


**Supplementary Figure 6** TOCSY of Reb D.


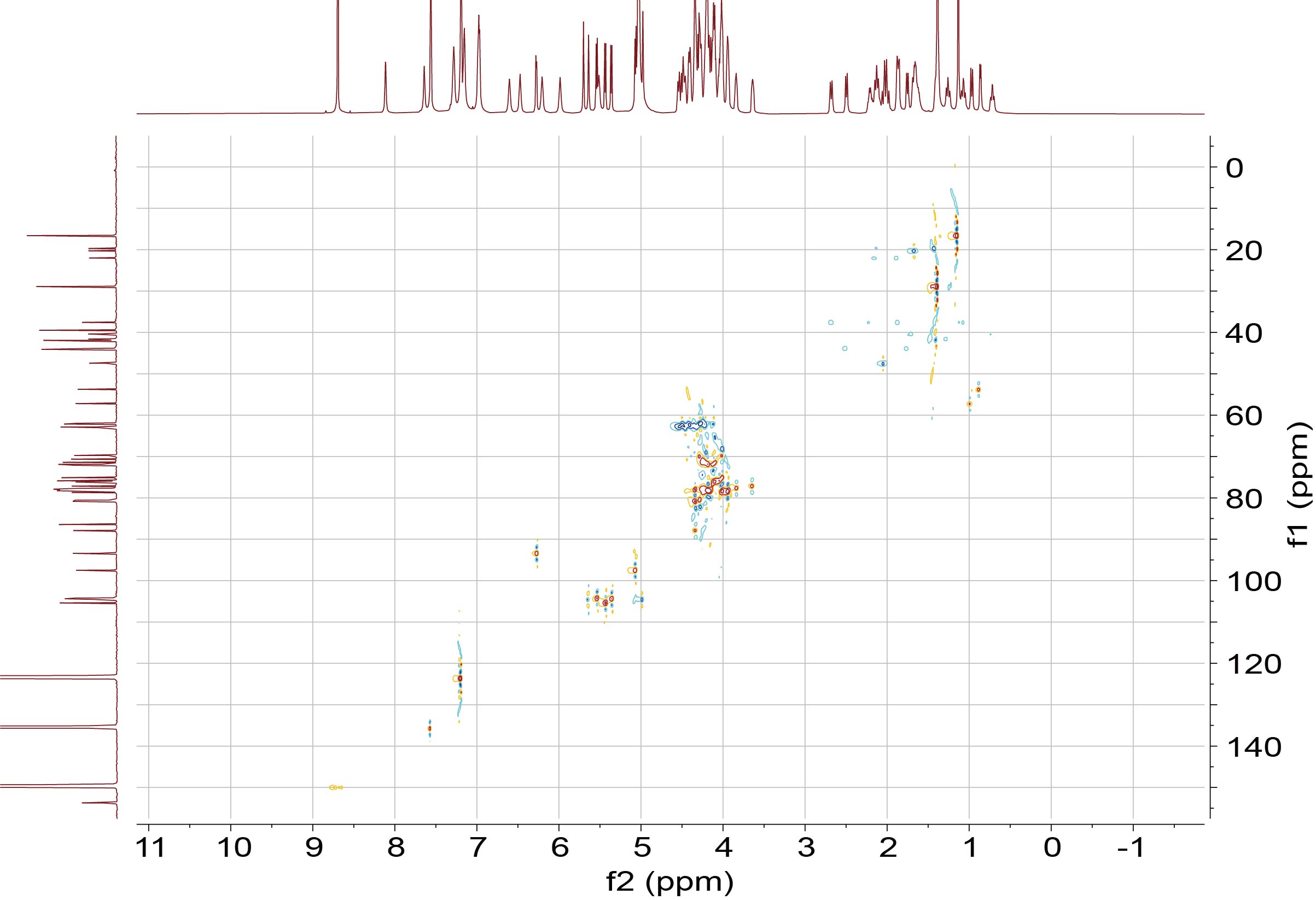


**Supplementary Figure 7** HSQC of Reb D.


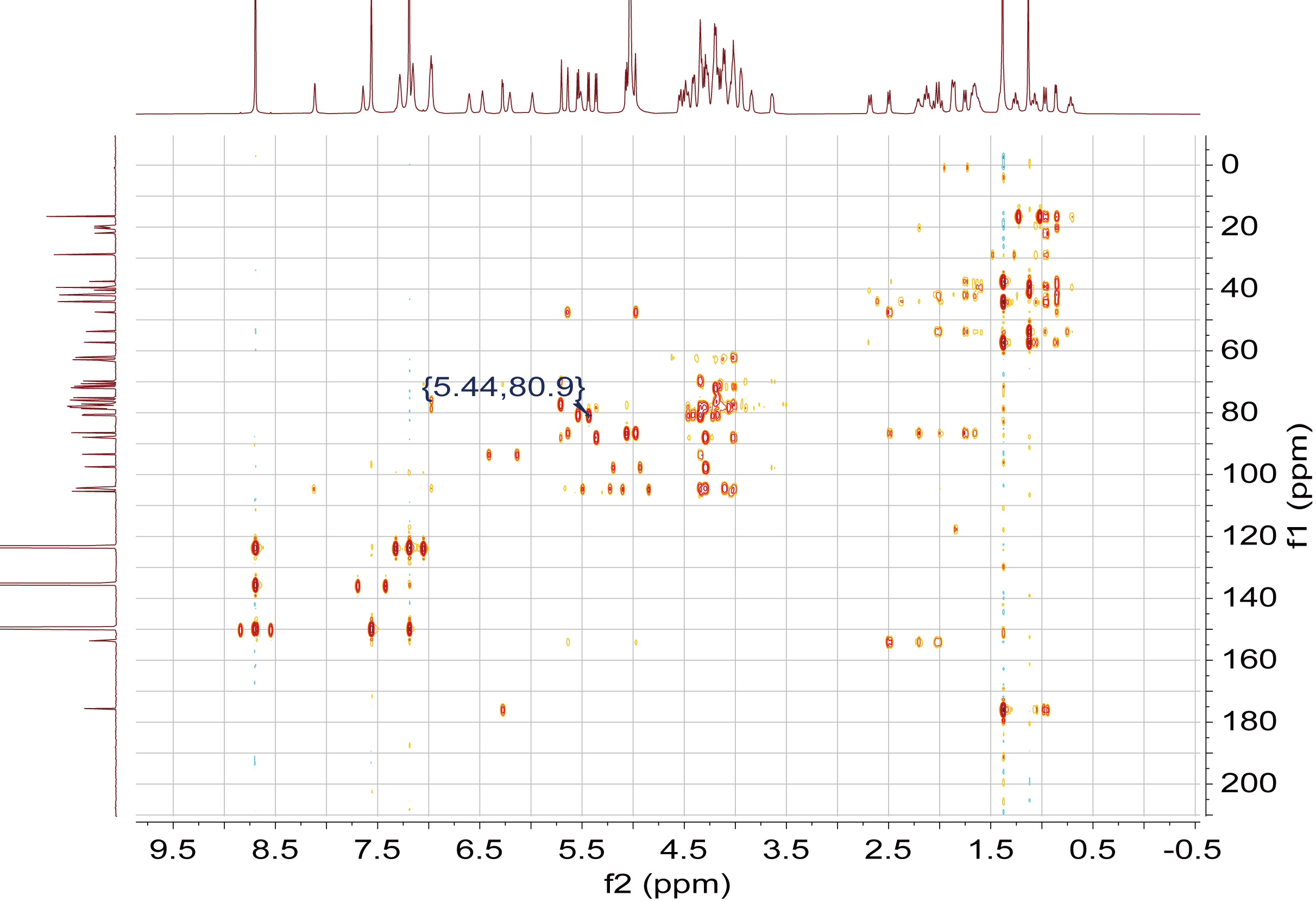


**Supplementary Figure 8** HMBC of Reb D.


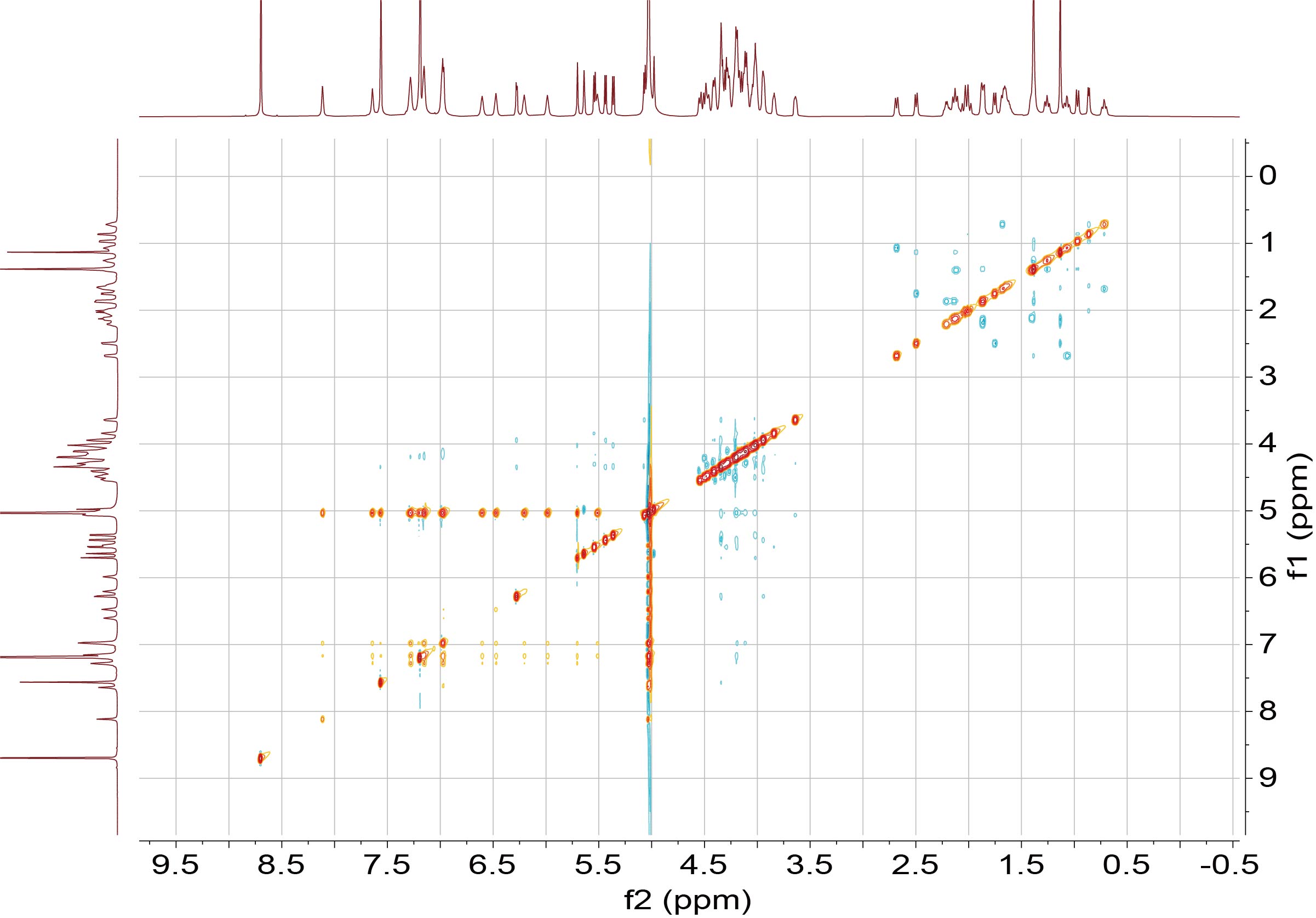


**Supplementary Figure 9** ROESY of Reb D.


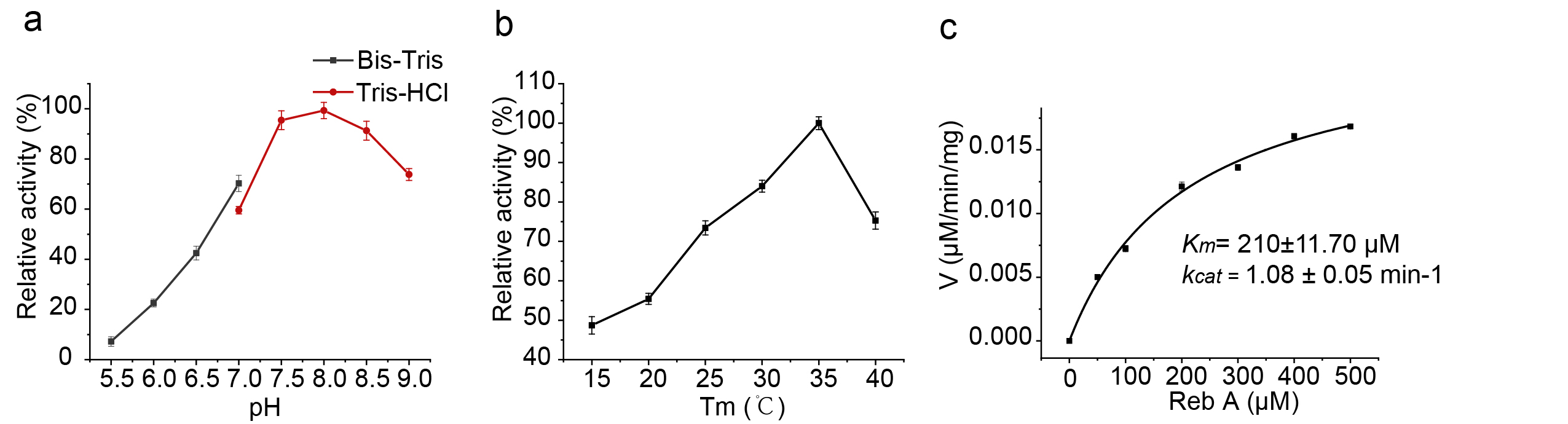


**Supplementary Figure 10** Enzymatic parameters of YojK. (a) Optimization of pH. (b) Optimization of reaction temperature. (c) The kinetic plots of YojK. Error bars mean standard deviation of three repeats.


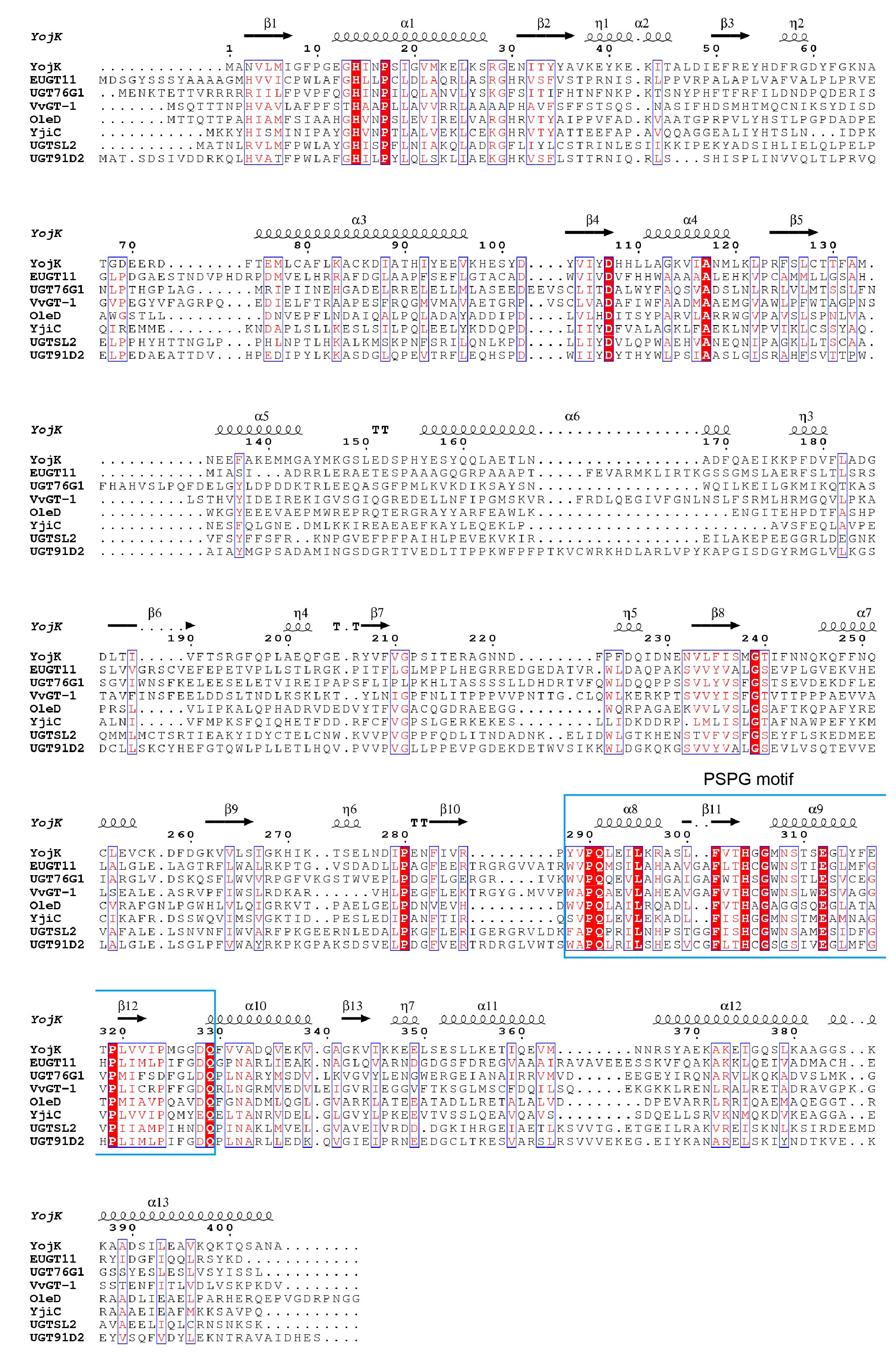


**Supplementary Figure 11** Multiple alignment of YojK with other UDP-glycosyltransferases (UGTs). EUGT11 from *Oryza sativa* (accession number: AAS07253.1), UGT76G1 from *Stevia rebaudiana* (accession number: Q6VAB4.1), VvGT-1 from *Vitis vinifera* (accession number: 2C1X_A), OleD from *Streptomyces antibioticus* (accession number: 2IYF_A), YjiC from *Bacillus* (accession number: WP_003232783.1), UGTSL2 from *Solanum lycopersicum* (accession number: XP_004250485.1), UGT91D2 from *Stevia rebaudiana* (accession number: B3VI56.1) were selected for alignment with YojK. The conserved region of plant secondary product glycosyltransferases (PSPG motif) was shown in blue box.


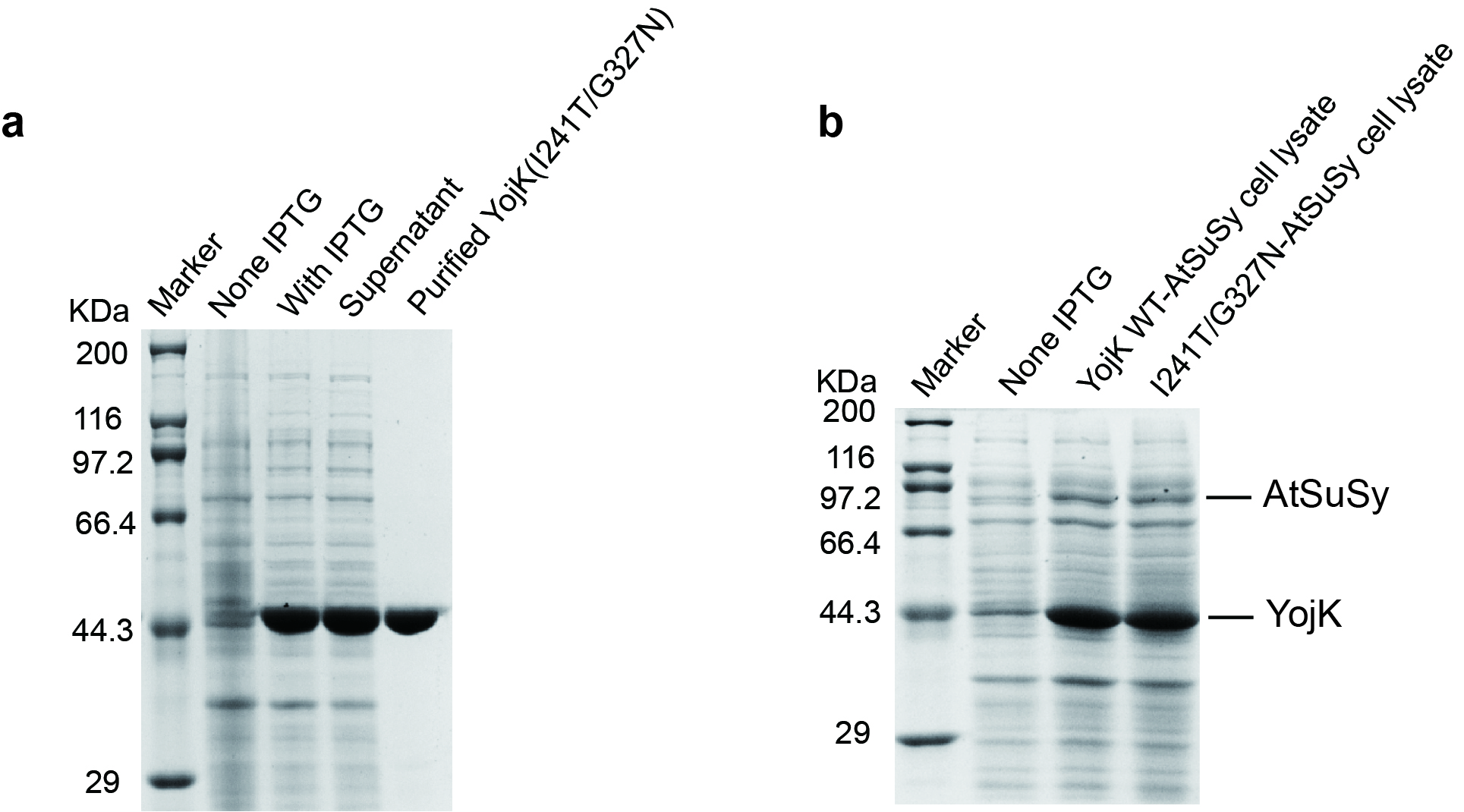


**Supplementary Figure 12** SDS-PAGE analysis of protein expression. (a) SDS-PAGE analysis of the heterologous expression of YojK-I241T/G327N. (b) SDS-PAGE analysis of heterologous co-expression of YojK or YojK-I241T/G327N coupled with *At*SuSy.
